# Supplementary material for: Comparative genomics provides new insights into the diversity, physiology, and sexuality of the only industrially exploited tremellomycete: Phaffia rhodozyma
Source: BMC Genomics. 2016 Nov 9;17:901. doi: 10.1186/s12864-016-3244-7 (PMC5103461; doi:10.1186/s12864-016-3244-7)
Supplement: Additional file 6: — List of orphan genes with links to PFAM (related to Additional file 1: Table S1). (ZIP 1428 kb) [file 12864_2016_3244_MOESM6_ESM.zip › BLAST_HTML_FTR/G02422_P.html]

BLAST Search Results


```
BLASTP 2.2.27+


Reference:
Stephen F. Altschul, Thomas L. Madden, Alejandro A. Schäffer,
Jinghui Zhang, Zheng Zhang, Webb Miller, and David J. Lipman (1997),
"Gapped BLAST and PSI-BLAST: a new generation of protein database
search programs", Nucleic Acids Res. 25:3389-3402.


Reference for
composition-based statistics:
Alejandro A. Schäffer, L. Aravind, Thomas L. Madden, Sergei
Shavirin, John L. Spouge, Yuri I. Wolf, Eugene V. Koonin, and
Stephen F. Altschul (2001), "Improving the accuracy of PSI-BLAST
protein database searches with composition-based statistics and
other refinements", Nucleic Acids Res. 29:2994-3005.


Database: nr
           71,551,133 sequences; 26,053,659,533 total letters


Query= G02422_P

Length=657
                                                                      Score     E
Sequences producing significant alignments:                          (Bits)  Value

emb|CED84678.1|  RNA recognition motif domain [Xanthophyllomyces ...  1334    0.0  
ref|XP_013103148.1|  PREDICTED: sex-lethal homolog isoform X1 [St...  45.4    0.13 
ref|XP_013103157.1|  PREDICTED: sex-lethal homolog isoform X5 [St...  45.4    0.14 
ref|XP_013103155.1|  PREDICTED: sex-lethal homolog isoform X3 [St...  45.1    0.15 
ref|XP_013103159.1|  PREDICTED: sex-lethal homolog isoform X6 [St...  45.1    0.16 
ref|XP_009164157.1|  hypothetical protein T265_12858, partial [Op...  45.4    0.17 
dbj|GAA29907.1|  ELAV like protein 2/3/4 [Clonorchis sinensis]        45.4    0.17 
ref|XP_013103154.1|  PREDICTED: sex-lethal homolog isoform X2 [St...  45.1    0.19 
ref|XP_013103156.1|  PREDICTED: sex-lethal homolog isoform X4 [St...  44.7    0.21 
gb|ALC48705.1|  Sxl [Drosophila busckii]                              44.7    0.21 
gb|KMZ08576.1|  LOW QUALITY PROTEIN: Sex lethal [Drosophila simul...  45.4    0.22 
ref|XP_002043208.1|  Sxl [Drosophila sechellia] >gb|EDW49349.1| S...  44.7    0.23 
ref|XP_002056776.1|  Sex lethal [Drosophila virilis] >gb|EDW62262...  44.7    0.25 
gb|ACX32993.1|  RE12578p [Drosophila melanogaster]                    44.7    0.25 
ref|XP_005809118.1|  PREDICTED: ribonucleoprotein PTB-binding 1-l...  45.1    0.25 
pdb|1B7F|A  Chain A, Sxl-Lethal ProteinRNA COMPLEX >pdb|1B7F|B Ch...  43.1    0.25 
ref|NP_001162686.1|  Sex lethal, isoform P [Drosophila melanogast...  44.7    0.26 
ref|XP_002067476.1|  GK16445 [Drosophila willistoni] >gb|EDW78462...  44.7    0.28 
ref|XP_011296428.1|  PREDICTED: sex-lethal homolog isoform X1 [Mu...  44.3    0.28 
ref|NP_001027065.1|  Sex lethal, isoform L [Drosophila melanogast...  44.3    0.29 
ref|XP_001963988.1|  GF20968 [Drosophila ananassae] >gb|EDV34437....  44.7    0.29 
ref|XP_002010681.1|  GI21573 [Drosophila mojavensis] >gb|EDW06336...  44.3    0.29 
ref|XP_011296432.1|  PREDICTED: sex-lethal homolog isoform X5 [Mu...  44.3    0.30 
ref|XP_001992493.1|  GH24172 [Drosophila grimshawi] >gb|EDV92200....  44.3    0.30 
ref|NP_001259304.1|  Sex lethal, isoform Y [Drosophila melanogast...  45.1    0.30 
ref|NP_727160.2|  Sex lethal, isoform J [Drosophila melanogaster]...  44.3    0.31 
gb|AAB81985.1|  Sex-lethal protein [Musca domestica]                  44.3    0.31 
ref|NP_727161.3|  Sex lethal, isoform W [Drosophila melanogaster]...  44.3    0.31 
gb|ADX35950.1|  SD08075p [Drosophila melanogaster]                    44.3    0.32 
ref|XP_011296430.1|  PREDICTED: sex-lethal homolog isoform X3 [Mu...  44.3    0.33 
ref|NP_001027063.1|  Sex lethal, isoform N [Drosophila melanogast...  44.3    0.33 
ref|XP_011296433.1|  PREDICTED: sex-lethal homolog isoform X7 [Mu...  44.3    0.33 
ref|NP_001259303.1|  Sex lethal, isoform X [Drosophila melanogast...  44.7    0.33 
ref|NP_001162688.1|  Sex lethal, isoform R [Drosophila melanogast...  44.3    0.33 
ref|NP_001162689.1|  Sex lethal, isoform AB [Drosophila melanogas...  44.3    0.34 
ref|XP_002106359.1|  Sex lethal [Drosophila simulans] >gb|EDX1731...  43.9    0.35 
ref|XP_011296429.1|  PREDICTED: sex-lethal homolog isoform X2 [Mu...  44.3    0.35 
ref|NP_727164.2|  Sex lethal, isoform H [Drosophila melanogaster]...  44.3    0.35 
ref|XP_005177313.1|  PREDICTED: sex-lethal homolog isoform X6 [Mu...  43.9    0.35 
gb|ACY56908.1|  RH48596p [Drosophila melanogaster]                    44.3    0.35 
pir||B39725  sex-lethal sex determination protein MS11 - fruit fl...  44.3    0.36 
gb|ADD20766.1|  RH41387p [Drosophila melanogaster]                    43.9    0.36 
sp|Q24668|SXL_DROSU  RecName: Full=Protein sex-lethal [Drosophila...  44.3    0.36 
ref|NP_001027062.1|  Sex lethal, isoform O [Drosophila melanogast...  44.3    0.37 
pdb|4QQB|A  Chain A, Structural Basis For The Assembly Of The Sxl...  42.7    0.39 
ref|NP_727163.2|  Sex lethal, isoform E [Drosophila melanogaster]...  43.9    0.41 
ref|XP_002025140.1|  GL26885 [Drosophila persimilis] >ref|XP_0021...  43.9    0.41 
ref|XP_011296431.1|  PREDICTED: sex-lethal homolog isoform X4 [Mu...  43.9    0.41 
emb|CAA67016.1|  sex-lethal [Drosophila subobscura]                   43.9    0.42 
ref|XP_001978518.1|  GG17637 [Drosophila erecta] >ref|XP_00210129...  43.5    0.51 
ref|NP_001274464.1|  sex-lethal homolog [Musca domestica] >sp|O17...  43.5    0.54 
gb|AAK20026.1|AF234184_1  sex-lethal protein SXL2 [Lucilia cuprina]   43.1    0.66 
gb|AAK20025.1|AF234183_1  sex-lethal protein SXL1 [Lucilia cuprina]   43.1    0.70 
sp|O97018|SXL_CHRRU  RecName: Full=Sex-lethal homolog [Chrysomya ...  43.1    0.70 
emb|CDS18761.1|  ELAV protein 2 [Echinococcus granulosus]             43.5    0.73 
emb|CDJ01832.1|  ELAV protein 2 [Echinococcus multilocularis] >em...  43.1    0.81 
gb|EUB55408.1|  ELAV-like protein [Echinococcus granulosus]           43.1    0.91 
gb|AAS45603.1|  sex-lethal [Bradysia coprophila]                      42.4    0.99 
sp|O61374|SXL_CERCA  RecName: Full=Sex-lethal homolog; AltName: F...  42.7    1.1  
ref|XP_012158510.1|  PREDICTED: sex-lethal homolog isoform X4 [Ce...  42.4    1.2  
ref|XP_008414224.1|  PREDICTED: ribonucleoprotein PTB-binding 1 i...  43.1    1.2  
ref|XP_012158507.1|  PREDICTED: sex-lethal homolog isoform X1 [Ce...  42.4    1.2  
ref|XP_011176864.1|  PREDICTED: sex-lethal homolog isoform X4 [Ba...  42.4    1.2  
ref|XP_012158508.1|  PREDICTED: sex-lethal homolog isoform X2 [Ce...  42.4    1.3  
pdb|3SXL|A  Chain A, Sex-Lethal Rna Recognition Domains 1 And 2 F...  41.2    1.3  
ref|XP_008414223.1|  PREDICTED: ribonucleoprotein PTB-binding 1 i...  42.7    1.3  
ref|XP_009903515.1|  PREDICTED: ELAV-like protein 2 isoform X1 [P...  42.4    1.3  
ref|XP_012158509.1|  PREDICTED: sex-lethal homolog isoform X3 [Ce...  42.4    1.4  
gb|KNC25619.1|  hypothetical protein FF38_03391 [Lucilia cuprina]     42.4    1.4  
gb|AIK25413.1|  sex-lethal-like protein, partial [Bactrocera tryoni]  42.4    1.4  
ref|XP_011176866.1|  PREDICTED: sex-lethal homolog isoform X6 [Ba...  42.4    1.4  
ref|XP_009903516.1|  PREDICTED: ELAV-like protein 2 isoform X2 [P...  42.4    1.5  
ref|XP_009685071.1|  PREDICTED: ELAV-like protein 2 isoform X3 [S...  42.0    1.6  
ref|NP_001266336.1|  sex-lethal homolog [Ceratitis capitata] >gb|...  42.0    1.6  
ref|XP_011176865.1|  PREDICTED: sex-lethal homolog isoform X5 [Ba...  42.0    1.6  
ref|XP_011176863.1|  PREDICTED: sex-lethal homolog isoform X3 [Ba...  42.0    1.8  
ref|XP_013795280.1|  PREDICTED: ELAV-like protein 2 isoform X1 [A...  41.6    2.0  
ref|XP_004391060.1|  PREDICTED: uncharacterized protein C6orf10 h...  41.6    2.2  
emb|CAG29242.1|  sex-lethal protein [Bactrocera oleae]                41.6    2.4  
ref|XP_013795281.1|  PREDICTED: ELAV-like protein 2 isoform X2 [A...  41.6    2.5  
ref|XP_013795282.1|  PREDICTED: ELAV-like protein 2 isoform X3 [A...  41.6    2.5  
ref|WP_048476863.1|  peptidase M28 [Streptomyces roseus] >gb|KMO9...  42.0    2.7  
ref|XP_009685069.1|  PREDICTED: ELAV-like protein 2 isoform X1 [S...  41.6    2.7  
ref|XP_009685070.1|  PREDICTED: ELAV-like protein 2 isoform X2 [S...  41.2    2.8  
ref|XP_011211864.1|  PREDICTED: sex-lethal homolog isoform X1 [Ba...  41.2    2.9  
ref|XP_011176861.1|  PREDICTED: sex-lethal homolog isoform X1 [Ba...  41.2    2.9  
ref|XP_009685073.1|  PREDICTED: ELAV-like protein 2 isoform X4 [S...  41.2    2.9  
ref|WP_026220098.1|  peptidase M28 [Streptomyces vitaminophilus]      41.6    3.2  
ref|XP_007560453.1|  PREDICTED: ribonucleoprotein PTB-binding 1 i...  41.6    3.3  
ref|XP_007560454.1|  PREDICTED: ribonucleoprotein PTB-binding 1 i...  41.6    3.3  
ref|XP_011176862.1|  PREDICTED: sex-lethal homolog isoform X2 [Ba...  41.2    3.5  
gb|AIK25412.1|  sex-lethal-like protein, partial [Bactrocera jarv...  40.8    3.5  
ref|XP_011211868.1|  PREDICTED: sex-lethal homolog isoform X3 [Ba...  40.8    3.6  
ref|XP_011211869.1|  PREDICTED: sex-lethal homolog isoform X4 [Ba...  40.8    3.6  
emb|CDS26875.1|  ELAV protein 2 [Hymenolepis microstoma]              41.2    4.1  
ref|XP_011211865.1|  PREDICTED: sex-lethal homolog isoform X2 [Ba...  40.8    4.4  
ref|XP_010724453.1|  PREDICTED: ELAV-like protein 2 isoform X2 [M...  40.4    4.6  
ref|XP_010724452.1|  PREDICTED: ELAV-like protein 2 isoform X1 [M...  40.4    4.6  
ref|XP_011211870.1|  PREDICTED: sex-lethal homolog isoform X5 [Ba...  40.4    4.7  
ref|WP_023109419.1|  hypothetical protein [Pseudomonas aeruginosa...  38.5    5.1  
ref|XP_005499263.1|  PREDICTED: ELAV-like protein 2 isoform X3 [C...  40.4    5.2  
ref|XP_013225007.1|  PREDICTED: ELAV-like protein 2 isoform X1 [C...  40.4    5.3  
ref|XP_004949599.1|  PREDICTED: ELAV-like protein 2 isoform X8 [G...  40.4    5.5  
ref|XP_009965948.1|  PREDICTED: ELAV-like protein 2 isoform X3 [T...  40.4    5.5  
ref|XP_004949598.1|  PREDICTED: ELAV-like protein 2 isoform X7 [G...  40.4    5.8  
ref|XP_004949606.1|  PREDICTED: ELAV-like protein 2 isoform X15 [...  40.4    5.9  
ref|XP_013225008.1|  PREDICTED: ELAV-like protein 2 isoform X2 [C...  40.4    6.0  
ref|XP_004949600.1|  PREDICTED: ELAV-like protein 2 isoform X9 [G...  40.4    6.2  
ref|XP_013225013.1|  PREDICTED: ELAV-like protein 2 isoform X4 [C...  40.4    6.2  
ref|XP_013225015.1|  PREDICTED: ELAV-like protein 2 isoform X5 [C...  40.0    6.5  
ref|XP_009965947.1|  PREDICTED: ELAV-like protein 2 isoform X2 [T...  40.0    6.5  
ref|XP_004949595.1|  PREDICTED: ELAV-like protein 2 isoform X4 [G...  40.4    6.7  
ref|XP_004949594.1|  PREDICTED: ELAV-like protein 2 isoform X3 [G...  40.4    6.8  
ref|XP_009965945.1|  PREDICTED: ELAV-like protein 2 isoform X1 [T...  40.0    7.5  
ref|XP_004949596.1|  PREDICTED: ELAV-like protein 2 isoform X5 [G...  40.0    7.5  
ref|XP_009965949.1|  PREDICTED: ELAV-like protein 2 isoform X4 [T...  40.0    7.8  
ref|XP_004949605.1|  PREDICTED: ELAV-like protein 2 isoform X14 [...  40.0    7.8  
ref|XP_003643117.1|  PREDICTED: ELAV-like protein 2 isoformX2 [Ga...  40.0    7.9  
ref|XP_004949603.1|  PREDICTED: ELAV-like protein 2 isoform X12 [...  39.7    8.5  
ref|XP_004949601.1|  PREDICTED: ELAV-like protein 2 isoform X10 [...  39.7    8.6  
ref|XP_004949604.1|  PREDICTED: ELAV-like protein 2 isoform X13 [...  39.7    9.2  


 >emb|CED84678.1| RNA recognition motif domain [Xanthophyllomyces dendrorhous]
Length=676

 Score = 1334 bits (3452),  Expect = 0.0, Method: Compositional matrix adjust.
 Identities = 653/657 (99%), Positives = 654/657 (99%), Gaps = 1/657 (0%)

Query  1    MSSFR-LRVLQERPYTSRPLVGPHRPPSLDDLELDPIDQDDSARASPIFVNRPPPFDKND  59
              +FR LRVLQERPYTSRPLVGPHRPPSLDDLELDPIDQDDSARASPIFVNRPPPFDKND
Sbjct  20   FGTFRSLRVLQERPYTSRPLVGPHRPPSLDDLELDPIDQDDSARASPIFVNRPPPFDKND  79

Query  60   YLLPSMVNKTEPIRNSRSSEASSRHPTSYTPWNKSNDKHIASAPLNTNNVSEEKARELCN  119
            YLLPSMVNKTEPIRNSRSSEASSRHPTSYTPWNKSNDKHIASAPLNTNNVSEEKARELCN
Sbjct  80   YLLPSMVNKTEPIRNSRSSEASSRHPTSYTPWNKSNDKHIASAPLNTNNVSEEKARELCN  139

Query  120  ILIEHLPPQMTKRELKALFNELDIRVNSIVKIQNGRAFVALNGEHSLQRILALDGNLTLM  179
            ILIEHLPPQMTKRELKALFNELDIRVNSIVKIQNGRAFVALNGEHSLQRILALDGNLTLM
Sbjct  140  ILIEHLPPQMTKRELKALFNELDIRVNSIVKIQNGRAFVALNGEHSLQRILALDGNLTLM  199

Query  180  QKKVLVTQIGPLKSSWRFTMDLPRDVTPEDIKKVLQPLNVDILYNKKLPVGEPNGDKRVL  239
            QKKVLVTQIGPLKSSWRFTMDLPRDVTPEDIKKVLQPLNVDILYNKKLPVGEPNGDKRVL
Sbjct  200  QKKVLVTQIGPLKSSWRFTMDLPRDVTPEDIKKVLQPLNVDILYNKKLPVGEPNGDKRVL  259

Query  240  QNDKGFLSRRSHRTSIRDSPNLSYGIRKYSTNVSVEEDDEFLRKGRRVRRPTEDIGVDPG  299
            QNDKGFLSRRSHRTSIRDSPNLSYGIRKYSTNVSVEEDDEFLRKGRRVRRPTEDIGVDPG
Sbjct  260  QNDKGFLSRRSHRTSIRDSPNLSYGIRKYSTNVSVEEDDEFLRKGRRVRRPTEDIGVDPG  319

Query  300  PLSEDSPRSSTNHPSSEESPAQHSEIPTSAFSTDTNLTKPDPPSSWESINNSVFSSNSEP  359
            PLSEDSPRSSTNHPSSEESPAQHSEIPTSAFSTDTNLTKPDPPSSWESINNSVFSSNSEP
Sbjct  320  PLSEDSPRSSTNHPSSEESPAQHSEIPTSAFSTDTNLTKPDPPSSWESINNSVFSSNSEP  379

Query  360  TGTTTEEERLVLKRVLAEALKYGKNKKAKSDVTSVAELGYEEYPAKSENVIMPADDSNDD  419
            TGTTTEEERLVLKRVLAEALKYGKNKKAKSDVTSVAELGYEEYPAKSENVIMPADDSNDD
Sbjct  380  TGTTTEEERLVLKRVLAEALKYGKNKKAKSDVTSVAELGYEEYPAKSENVIMPADDSNDD  439

Query  420  KAFDQLTIEAEQRLHLEKIRDDNLIYQLRVDGFGSQWRYKDLMSLIQEYEIDCSIPTGPQ  479
            KAFDQLTIEAEQRLHLEKIRDDNLIYQLRVDGFGSQWRYKDLMSLIQEYEIDCSIPTGPQ
Sbjct  440  KAFDQLTIEAEQRLHLEKIRDDNLIYQLRVDGFGSQWRYKDLMSLIQEYEIDCSIPTGPQ  499

Query  480  NGLVLMSVVGKHRFEKLFRMAGLEMLLKLTLISCSTTVADLLVALEMAQLKRDHLLENLG  539
            NGLVLMSVVGKHRFEKLFRMAGLEMLLKLTLISCSTTVADLLVALEMAQLKRDHLLENLG
Sbjct  500  NGLVLMSVVGKHRFEKLFRMAGLEMLLKLTLISCSTTVADLLVALEMAQLKRDHLLENLG  559

Query  540  INIGKIWTPMSSDGRKSSPSSTQSKNPLQMIVPQYRPNTDQKHYAVHPDAVLNNPPTVPY  599
            INIGKIWTPMSSDGRKSSPSSTQSKNPLQMIVPQYRPNTDQKHYAVHPDAVLNNPPTVPY
Sbjct  560  INIGKIWTPMSSDGRKSSPSSTQSKNPLQMIVPQYRPNTDQKHYAVHPDAVLNNPPTVPY  619

Query  600  HPPFLFPDDDPHFGRLKEGNKRFKSNETKNPRKPISGHSAEYTKSDNSKSDSLHKDR  656
            HPPFLFPDDDPHFGRLKEGNKRFKSNETKNPRKPISGHSAEYTKSDNSKSDSLHKDR
Sbjct  620  HPPFLFPDDDPHFGRLKEGNKRFKSNETKNPRKPISGHSAEYTKSDNSKSDSLHKDR  676


>ref|XP_013103148.1| PREDICTED: sex-lethal homolog isoform X1 [Stomoxys calcitrans]
 ref|XP_013103149.1| PREDICTED: sex-lethal homolog isoform X1 [Stomoxys calcitrans]
 ref|XP_013103150.1| PREDICTED: sex-lethal homolog isoform X1 [Stomoxys calcitrans]
 ref|XP_013103151.1| PREDICTED: sex-lethal homolog isoform X1 [Stomoxys calcitrans]
 ref|XP_013103152.1| PREDICTED: sex-lethal homolog isoform X1 [Stomoxys calcitrans]
 ref|XP_013103153.1| PREDICTED: sex-lethal homolog isoform X1 [Stomoxys calcitrans]
Length=334

 Score = 45.4 bits (106),  Expect = 0.13, Method: Compositional matrix adjust.
 Identities = 29/106 (27%), Positives = 50/106 (47%), Gaps = 10/106 (9%)

Query  119  NILIEHLPPQMTKRELKALF------NELDIRVNSIVKIQNGRAFVALNGEHSLQRILAL  172
            N+++ +LP  MT REL ALF      N   I  +       G AFV    E   Q  +  
Sbjct  112  NLIVNYLPQDMTDRELYALFRTCGPINTCRIMKDYKTGYSFGYAFVDFASEIDAQNAIKT  171

Query  173  DGNLTLMQKKVLVTQIGP----LKSSWRFTMDLPRDVTPEDIKKVL  214
               +T+  K++ V+   P    +K +  +  +LPR +T ++++K+ 
Sbjct  172  VNGITVRNKRIKVSYARPGGESIKDTNLYVTNLPRTITDDELEKIF  217


>ref|XP_013103157.1| PREDICTED: sex-lethal homolog isoform X5 [Stomoxys calcitrans]
Length=315

 Score = 45.4 bits (106),  Expect = 0.14, Method: Compositional matrix adjust.
 Identities = 29/106 (27%), Positives = 50/106 (47%), Gaps = 10/106 (9%)

Query  119  NILIEHLPPQMTKRELKALF------NELDIRVNSIVKIQNGRAFVALNGEHSLQRILAL  172
            N+++ +LP  MT REL ALF      N   I  +       G AFV    E   Q  +  
Sbjct  93   NLIVNYLPQDMTDRELYALFRTCGPINTCRIMKDYKTGYSFGYAFVDFASEIDAQNAIKT  152

Query  173  DGNLTLMQKKVLVTQIGP----LKSSWRFTMDLPRDVTPEDIKKVL  214
               +T+  K++ V+   P    +K +  +  +LPR +T ++++K+ 
Sbjct  153  VNGITVRNKRIKVSYARPGGESIKDTNLYVTNLPRTITDDELEKIF  198


>ref|XP_013103155.1| PREDICTED: sex-lethal homolog isoform X3 [Stomoxys calcitrans]
Length=326

 Score = 45.1 bits (105),  Expect = 0.15, Method: Compositional matrix adjust.
 Identities = 29/106 (27%), Positives = 50/106 (47%), Gaps = 10/106 (9%)

Query  119  NILIEHLPPQMTKRELKALF------NELDIRVNSIVKIQNGRAFVALNGEHSLQRILAL  172
            N+++ +LP  MT REL ALF      N   I  +       G AFV    E   Q  +  
Sbjct  104  NLIVNYLPQDMTDRELYALFRTCGPINTCRIMKDYKTGYSFGYAFVDFASEIDAQNAIKT  163

Query  173  DGNLTLMQKKVLVTQIGP----LKSSWRFTMDLPRDVTPEDIKKVL  214
               +T+  K++ V+   P    +K +  +  +LPR +T ++++K+ 
Sbjct  164  VNGITVRNKRIKVSYARPGGESIKDTNLYVTNLPRTITDDELEKIF  209


>ref|XP_013103159.1| PREDICTED: sex-lethal homolog isoform X6 [Stomoxys calcitrans]
Length=307

 Score = 45.1 bits (105),  Expect = 0.16, Method: Compositional matrix adjust.
 Identities = 29/106 (27%), Positives = 50/106 (47%), Gaps = 10/106 (9%)

Query  119  NILIEHLPPQMTKRELKALF------NELDIRVNSIVKIQNGRAFVALNGEHSLQRILAL  172
            N+++ +LP  MT REL ALF      N   I  +       G AFV    E   Q  +  
Sbjct  85   NLIVNYLPQDMTDRELYALFRTCGPINTCRIMKDYKTGYSFGYAFVDFASEIDAQNAIKT  144

Query  173  DGNLTLMQKKVLVTQIGP----LKSSWRFTMDLPRDVTPEDIKKVL  214
               +T+  K++ V+   P    +K +  +  +LPR +T ++++K+ 
Sbjct  145  VNGITVRNKRIKVSYARPGGESIKDTNLYVTNLPRTITDDELEKIF  190


>ref|XP_009164157.1| hypothetical protein T265_12858, partial [Opisthorchis viverrini]
 gb|KER32143.1| hypothetical protein T265_12858, partial [Opisthorchis viverrini]
Length=645

 Score = 45.4 bits (106),  Expect = 0.17, Method: Compositional matrix adjust.
 Identities = 35/140 (25%), Positives = 64/140 (46%), Gaps = 14/140 (10%)

Query  101  SAPLNTNNVSEEKARELCNILIEHLPPQMTKRELKALFNEL-DIRVNSIVKIQN-----G  154
            S P+N   V+        N++I +LPP M++ E++ALF+ + ++    +V+ +      G
Sbjct  103  SGPINNTAVTNSPPENKTNLIINYLPPNMSQEEVRALFSSIGEVESCKLVREKTSGESLG  162

Query  155  RAFVALNGEHSLQRILALDGNLTLMQKKVLVTQIGP----LKSSWRFTMDLPRDVTPEDI  210
             AFV         + +     L L  K V V+   P    +K +  +   LPR +T  ++
Sbjct  163  YAFVKFYDPVDAGKAIKTLNGLRLQNKTVKVSLARPSSEAIKGANLYICGLPRKMTQPEL  222

Query  211  KKVLQP----LNVDILYNKK  226
            +K+       +   ILY+ K
Sbjct  223  EKLFSACGHIITARILYDTK  242


>dbj|GAA29907.1| ELAV like protein 2/3/4 [Clonorchis sinensis]
Length=645

 Score = 45.4 bits (106),  Expect = 0.17, Method: Compositional matrix adjust.
 Identities = 35/140 (25%), Positives = 64/140 (46%), Gaps = 14/140 (10%)

Query  101  SAPLNTNNVSEEKARELCNILIEHLPPQMTKRELKALFNEL-DIRVNSIVKIQN-----G  154
            S P+N   V+        N++I +LPP M++ E++ALF+ + ++    +V+ +      G
Sbjct  103  SGPVNNTAVTNSPPENKTNLIINYLPPNMSQEEVRALFSSIGEVESCKLVREKTSGESLG  162

Query  155  RAFVALNGEHSLQRILALDGNLTLMQKKVLVTQIGP----LKSSWRFTMDLPRDVTPEDI  210
             AFV         + +     L L  K V V+   P    +K +  +   LPR +T  ++
Sbjct  163  YAFVKFYDPLDAGKAIKTLNGLRLQNKTVKVSLARPSSEAIKGANLYICGLPRKMTQPEL  222

Query  211  KKVLQP----LNVDILYNKK  226
            +K+       +   ILY+ K
Sbjct  223  EKLFSACGHIITARILYDTK  242


>ref|XP_013103154.1| PREDICTED: sex-lethal homolog isoform X2 [Stomoxys calcitrans]
Length=333

 Score = 45.1 bits (105),  Expect = 0.19, Method: Compositional matrix adjust.
 Identities = 29/106 (27%), Positives = 50/106 (47%), Gaps = 10/106 (9%)

Query  119  NILIEHLPPQMTKRELKALF------NELDIRVNSIVKIQNGRAFVALNGEHSLQRILAL  172
            N+++ +LP  MT REL ALF      N   I  +       G AFV    E   Q  +  
Sbjct  112  NLIVNYLPQDMTDRELYALFRTCGPINTCRIMKDYKTGYSFGYAFVDFASEIDAQNAIKT  171

Query  173  DGNLTLMQKKVLVTQIGP----LKSSWRFTMDLPRDVTPEDIKKVL  214
               +T+  K++ V+   P    +K +  +  +LPR +T ++++K+ 
Sbjct  172  VNGITVRNKRIKVSYARPGGESIKDTNLYVTNLPRTITDDELEKIF  217


>ref|XP_013103156.1| PREDICTED: sex-lethal homolog isoform X4 [Stomoxys calcitrans]
Length=325

 Score = 44.7 bits (104),  Expect = 0.21, Method: Compositional matrix adjust.
 Identities = 29/106 (27%), Positives = 50/106 (47%), Gaps = 10/106 (9%)

Query  119  NILIEHLPPQMTKRELKALF------NELDIRVNSIVKIQNGRAFVALNGEHSLQRILAL  172
            N+++ +LP  MT REL ALF      N   I  +       G AFV    E   Q  +  
Sbjct  104  NLIVNYLPQDMTDRELYALFRTCGPINTCRIMKDYKTGYSFGYAFVDFASEIDAQNAIKT  163

Query  173  DGNLTLMQKKVLVTQIGP----LKSSWRFTMDLPRDVTPEDIKKVL  214
               +T+  K++ V+   P    +K +  +  +LPR +T ++++K+ 
Sbjct  164  VNGITVRNKRIKVSYARPGGESIKDTNLYVTNLPRTITDDELEKIF  209


>gb|ALC48705.1| Sxl [Drosophila busckii]
Length=327

 Score = 44.7 bits (104),  Expect = 0.21, Method: Compositional matrix adjust.
 Identities = 29/106 (27%), Positives = 50/106 (47%), Gaps = 10/106 (9%)

Query  119  NILIEHLPPQMTKRELKALF------NELDIRVNSIVKIQNGRAFVALNGEHSLQRILAL  172
            N+++ +LP  MT REL ALF      N   I  +       G AFV    E   QR + +
Sbjct  93   NLIVNYLPQDMTDRELYALFRAIGPINTCRIMRDYKTGYSFGYAFVDFTSEMDSQRAIKV  152

Query  173  DGNLTLMQKKVLVTQIGP----LKSSWRFTMDLPRDVTPEDIKKVL  214
               +T+  K++ V+   P    +K +  +  +LPR +T + ++ + 
Sbjct  153  LNGITVRNKRLKVSYARPGGESIKDTNLYVTNLPRTITDDQLETIF  198


>gb|KMZ08576.1| LOW QUALITY PROTEIN: Sex lethal [Drosophila simulans]
Length=721

 Score = 45.4 bits (106),  Expect = 0.22, Method: Compositional matrix adjust.
 Identities = 29/106 (27%), Positives = 49/106 (46%), Gaps = 10/106 (9%)

Query  119  NILIEHLPPQMTKRELKALF------NELDIRVNSIVKIQNGRAFVALNGEHSLQRILAL  172
            N+++ +LP  MT REL ALF      N   I  +       G AFV    E   QR + +
Sbjct  118  NLIVNYLPQDMTDRELYALFRAIGPINTCRIMRDYKTGYSFGYAFVDFTSEMDSQRAIKV  177

Query  173  DGNLTLMQKKVLVTQIGP----LKSSWRFTMDLPRDVTPEDIKKVL  214
               +T+  K++ V+   P    +K +  +  +LPR +T + +  + 
Sbjct  178  LNGITVRNKRLKVSYARPGGESIKDTNLYVTNLPRTITDDQLDTIF  223


>ref|XP_002043208.1| Sxl [Drosophila sechellia]
 gb|EDW49349.1| Sxl [Drosophila sechellia]
Length=301

 Score = 44.7 bits (104),  Expect = 0.23, Method: Compositional matrix adjust.
 Identities = 29/106 (27%), Positives = 49/106 (46%), Gaps = 10/106 (9%)

Query  119  NILIEHLPPQMTKRELKALF------NELDIRVNSIVKIQNGRAFVALNGEHSLQRILAL  172
            N+++ +LP  MT REL ALF      N   I  +       G AFV    E   QR + +
Sbjct  118  NLIVNYLPQDMTDRELYALFRAIGPINTCRIMRDYKTGYSFGYAFVDFTSEMDSQRAIKV  177

Query  173  DGNLTLMQKKVLVTQIGP----LKSSWRFTMDLPRDVTPEDIKKVL  214
               +T+  K++ V+   P    +K +  +  +LPR +T + +  + 
Sbjct  178  LNGITVRNKRLKVSYARPGGESIKDTNLYVTNLPRTITDDQLDTIF  223


>ref|XP_002056776.1| Sex lethal [Drosophila virilis]
 gb|EDW62262.1| Sex lethal [Drosophila virilis]
Length=368

 Score = 44.7 bits (104),  Expect = 0.25, Method: Compositional matrix adjust.
 Identities = 29/106 (27%), Positives = 49/106 (46%), Gaps = 10/106 (9%)

Query  119  NILIEHLPPQMTKRELKALF------NELDIRVNSIVKIQNGRAFVALNGEHSLQRILAL  172
            N+++ +LP  MT REL ALF      N   I  +       G AFV    E   QR + +
Sbjct  119  NLIVNYLPQDMTDRELYALFRAIGPINTCRIMRDYKTGYSFGYAFVDFTSEMDSQRAIKV  178

Query  173  DGNLTLMQKKVLVTQIGP----LKSSWRFTMDLPRDVTPEDIKKVL  214
               +T+  K++ V+   P    +K +  +  +LPR +T + +  + 
Sbjct  179  LNGITVRNKRLKVSYARPGGESIKDTNLYVTNLPRTITDDQLDTIF  224


>gb|ACX32993.1| RE12578p [Drosophila melanogaster]
Length=360

 Score = 44.7 bits (104),  Expect = 0.25, Method: Compositional matrix adjust.
 Identities = 29/106 (27%), Positives = 49/106 (46%), Gaps = 10/106 (9%)

Query  119  NILIEHLPPQMTKRELKALF------NELDIRVNSIVKIQNGRAFVALNGEHSLQRILAL  172
            N+++ +LP  MT REL ALF      N   I  +       G AFV    E   QR + +
Sbjct  132  NLIVNYLPQDMTDRELYALFRAIGPINTCRIMRDYKTGYSFGYAFVDFTSEMDSQRAIKV  191

Query  173  DGNLTLMQKKVLVTQIGP----LKSSWRFTMDLPRDVTPEDIKKVL  214
               +T+  K++ V+   P    +K +  +  +LPR +T + +  + 
Sbjct  192  LNGITVRNKRLKVSYARPGGESIKDTNLYVTNLPRTITDDQLDTIF  237


>ref|XP_005809118.1| PREDICTED: ribonucleoprotein PTB-binding 1-like [Xiphophorus 
maculatus]
Length=736

 Score = 45.1 bits (105),  Expect = 0.25, Method: Compositional matrix adjust.
 Identities = 46/186 (25%), Positives = 85/186 (46%), Gaps = 12/186 (6%)

Query  39   DDSARASPIFVNRPPPFDKNDYLLPSMVNKTEPIRNSRSSEASSRHPTSYTP--WNKSND  96
            D+ A + P FV+RP  F  N  L     N     R+     AS     +  P    + ++
Sbjct  14   DEGADSGPTFVSRP--FSSNHDLATDKENFMSGDRHRHPEFASVEEDATSGPECQRRVDE  71

Query  97   KHIASAPLNTNNVSEEKARELCN---ILIEHLPPQMTKRELKALFNELDIRVNSIVKIQN  153
              +  +P +  +  E   RE  N   I+I++LPP +T +E+  L    D++   + K + 
Sbjct  72   DLVPLSPEDIESRLERTRREFYNRRKIIIKNLPPDVTNQEVHELLGNYDLKYCFVDKYK-  130

Query  154  GRAFVA-LNGEHSLQRILALDGNLTLMQKKVLVTQIGPLKSSWRFTMDLPRDVTPEDIKK  212
            G AFV  LNGE +   I     +  +++++ +  Q+ P   +     +LPR  T +  ++
Sbjct  131  GTAFVTLLNGEQAQCAIKEF--HQIMLREREISVQLQP-TDTLLCIANLPRAFTQQQFEE  187

Query  213  VLQPLN  218
            +++P  
Sbjct  188  LVRPFG  193


>pdb|1B7F|A Chain A, Sxl-Lethal ProteinRNA COMPLEX
 pdb|1B7F|B Chain B, Sxl-Lethal ProteinRNA COMPLEX
Length=168

 Score = 43.1 bits (100),  Expect = 0.25, Method: Compositional matrix adjust.
 Identities = 29/106 (27%), Positives = 49/106 (46%), Gaps = 10/106 (9%)

Query  119  NILIEHLPPQMTKRELKALF------NELDIRVNSIVKIQNGRAFVALNGEHSLQRILAL  172
            N+++ +LP  MT REL ALF      N   I  +       G AFV    E   QR + +
Sbjct  5    NLIVNYLPQDMTDRELYALFRAIGPINTCRIMRDYKTGYSYGYAFVDFTSEMDSQRAIKV  64

Query  173  DGNLTLMQKKVLVTQIGP----LKSSWRFTMDLPRDVTPEDIKKVL  214
               +T+  K++ V+   P    +K +  +  +LPR +T + +  + 
Sbjct  65   LNGITVRNKRLKVSYARPGGESIKDTNLYVTNLPRTITDDQLDTIF  110


>ref|NP_001162686.1| Sex lethal, isoform P [Drosophila melanogaster]
 gb|AAO39587.1| LD15933p [Drosophila melanogaster]
 gb|ACZ95222.1| Sex lethal, isoform P [Drosophila melanogaster]
Length=352

 Score = 44.7 bits (104),  Expect = 0.26, Method: Compositional matrix adjust.
 Identities = 29/106 (27%), Positives = 49/106 (46%), Gaps = 10/106 (9%)

Query  119  NILIEHLPPQMTKRELKALF------NELDIRVNSIVKIQNGRAFVALNGEHSLQRILAL  172
            N+++ +LP  MT REL ALF      N   I  +       G AFV    E   QR + +
Sbjct  126  NLIVNYLPQDMTDRELYALFRAIGPINTCRIMRDYKTGYSFGYAFVDFTSEMDSQRAIKV  185

Query  173  DGNLTLMQKKVLVTQIGP----LKSSWRFTMDLPRDVTPEDIKKVL  214
               +T+  K++ V+   P    +K +  +  +LPR +T + +  + 
Sbjct  186  LNGITVRNKRLKVSYARPGGESIKDTNLYVTNLPRTITDDQLDTIF  231


>ref|XP_002067476.1| GK16445 [Drosophila willistoni]
 gb|EDW78462.1| GK16445 [Drosophila willistoni]
Length=373

 Score = 44.7 bits (104),  Expect = 0.28, Method: Compositional matrix adjust.
 Identities = 29/106 (27%), Positives = 49/106 (46%), Gaps = 10/106 (9%)

Query  119  NILIEHLPPQMTKRELKALF------NELDIRVNSIVKIQNGRAFVALNGEHSLQRILAL  172
            N+++ +LP  MT REL ALF      N   I  +       G AFV    E   QR + +
Sbjct  122  NLIVNYLPQDMTDRELYALFRAIGPINTCRIMRDYKTGYSFGYAFVDFTSEMDSQRAIKV  181

Query  173  DGNLTLMQKKVLVTQIGP----LKSSWRFTMDLPRDVTPEDIKKVL  214
               +T+  K++ V+   P    +K +  +  +LPR +T + +  + 
Sbjct  182  LNGITVRNKRLKVSYARPGGESIKDTNLYVTNLPRTITDDQLDTIF  227


>ref|XP_011296428.1| PREDICTED: sex-lethal homolog isoform X1 [Musca domestica]
Length=347

 Score = 44.3 bits (103),  Expect = 0.28, Method: Compositional matrix adjust.
 Identities = 29/106 (27%), Positives = 50/106 (47%), Gaps = 10/106 (9%)

Query  119  NILIEHLPPQMTKRELKALF------NELDIRVNSIVKIQNGRAFVALNGEHSLQRILAL  172
            N+++ +LP  MT REL ALF      N   I  +       G AFV    E   Q  +  
Sbjct  111  NLIVNYLPQDMTDRELYALFRTCGPINTCRIMKDYKTGYSFGYAFVDFASEIDAQNAIKT  170

Query  173  DGNLTLMQKKVLVTQIGP----LKSSWRFTMDLPRDVTPEDIKKVL  214
               +T+  K++ V+   P    +K +  +  +LPR +T ++++K+ 
Sbjct  171  VNGITVRNKRLKVSYARPGGESIKDTNLYVTNLPRTITDDELEKIF  216


>ref|NP_001027065.1| Sex lethal, isoform L [Drosophila melanogaster]
 ref|NP_727162.2| Sex lethal, isoform D [Drosophila melanogaster]
 ref|NP_001162690.1| Sex lethal, isoform T [Drosophila melanogaster]
 6 more sequence titles

sp|P19339|SXL_DROME RecName: Full=Protein sex-lethal
 gb|AAA28884.1| sex-linked protein [Drosophila melanogaster]
 gb|AAA28922.1| Sx1 [Drosophila melanogaster]
 gb|AAG22410.1| Sex lethal, isoform D [Drosophila melanogaster]
 gb|AAZ52511.1| Sex lethal, isoform L [Drosophila melanogaster]
 gb|ACZ95226.1| Sex lethal, isoform T [Drosophila melanogaster]

Length=354

 Score = 44.3 bits (103),  Expect = 0.29, Method: Compositional matrix adjust.
 Identities = 29/106 (27%), Positives = 49/106 (46%), Gaps = 10/106 (9%)

Query  119  NILIEHLPPQMTKRELKALF------NELDIRVNSIVKIQNGRAFVALNGEHSLQRILAL  172
            N+++ +LP  MT REL ALF      N   I  +       G AFV    E   QR + +
Sbjct  126  NLIVNYLPQDMTDRELYALFRAIGPINTCRIMRDYKTGYSFGYAFVDFTSEMDSQRAIKV  185

Query  173  DGNLTLMQKKVLVTQIGP----LKSSWRFTMDLPRDVTPEDIKKVL  214
               +T+  K++ V+   P    +K +  +  +LPR +T + +  + 
Sbjct  186  LNGITVRNKRLKVSYARPGGESIKDTNLYVTNLPRTITDDQLDTIF  231


>ref|XP_001963988.1| GF20968 [Drosophila ananassae]
 gb|EDV34437.1| GF20968 [Drosophila ananassae]
Length=379

 Score = 44.7 bits (104),  Expect = 0.29, Method: Compositional matrix adjust.
 Identities = 29/106 (27%), Positives = 49/106 (46%), Gaps = 10/106 (9%)

Query  119  NILIEHLPPQMTKRELKALF------NELDIRVNSIVKIQNGRAFVALNGEHSLQRILAL  172
            N+++ +LP  MT REL ALF      N   I  +       G AFV    E   QR + +
Sbjct  129  NLIVNYLPQDMTDRELYALFRAIGPINTCRIMRDYKTGYSFGYAFVDFTSEMDSQRAIKV  188

Query  173  DGNLTLMQKKVLVTQIGP----LKSSWRFTMDLPRDVTPEDIKKVL  214
               +T+  K++ V+   P    +K +  +  +LPR +T + +  + 
Sbjct  189  LNGITVRNKRLKVSYARPGGESIKDTNLYVTNLPRTITDDQLDTIF  234


>ref|XP_002010681.1| GI21573 [Drosophila mojavensis]
 gb|EDW06336.1| GI21573 [Drosophila mojavensis]
Length=367

 Score = 44.3 bits (103),  Expect = 0.29, Method: Compositional matrix adjust.
 Identities = 29/106 (27%), Positives = 49/106 (46%), Gaps = 10/106 (9%)

Query  119  NILIEHLPPQMTKRELKALF------NELDIRVNSIVKIQNGRAFVALNGEHSLQRILAL  172
            N+++ +LP  MT REL ALF      N   I  +       G AFV    E   QR + +
Sbjct  118  NLIVNYLPQDMTDRELYALFRAIGPINTCRIMRDYKTGYSFGYAFVDFTSEMDSQRAIKV  177

Query  173  DGNLTLMQKKVLVTQIGP----LKSSWRFTMDLPRDVTPEDIKKVL  214
               +T+  K++ V+   P    +K +  +  +LPR +T + +  + 
Sbjct  178  LNGITVRNKRLKVSYARPGGESIKDTNLYVTNLPRTITDDQLDTIF  223


>ref|XP_011296432.1| PREDICTED: sex-lethal homolog isoform X5 [Musca domestica]
Length=327

 Score = 44.3 bits (103),  Expect = 0.30, Method: Compositional matrix adjust.
 Identities = 29/106 (27%), Positives = 50/106 (47%), Gaps = 10/106 (9%)

Query  119  NILIEHLPPQMTKRELKALF------NELDIRVNSIVKIQNGRAFVALNGEHSLQRILAL  172
            N+++ +LP  MT REL ALF      N   I  +       G AFV    E   Q  +  
Sbjct  91   NLIVNYLPQDMTDRELYALFRTCGPINTCRIMKDYKTGYSFGYAFVDFASEIDAQNAIKT  150

Query  173  DGNLTLMQKKVLVTQIGP----LKSSWRFTMDLPRDVTPEDIKKVL  214
               +T+  K++ V+   P    +K +  +  +LPR +T ++++K+ 
Sbjct  151  VNGITVRNKRLKVSYARPGGESIKDTNLYVTNLPRTITDDELEKIF  196


>ref|XP_001992493.1| GH24172 [Drosophila grimshawi]
 gb|EDV92200.1| GH24172 [Drosophila grimshawi]
Length=365

 Score = 44.3 bits (103),  Expect = 0.30, Method: Compositional matrix adjust.
 Identities = 29/106 (27%), Positives = 49/106 (46%), Gaps = 10/106 (9%)

Query  119  NILIEHLPPQMTKRELKALF------NELDIRVNSIVKIQNGRAFVALNGEHSLQRILAL  172
            N+++ +LP  MT REL ALF      N   I  +       G AFV    E   QR + +
Sbjct  118  NLIVNYLPQDMTDRELYALFRSIGTINTCRIMRDYKTGYSFGYAFVDFTSEMDSQRAIKV  177

Query  173  DGNLTLMQKKVLVTQIGP----LKSSWRFTMDLPRDVTPEDIKKVL  214
               +T+  K++ V+   P    +K +  +  +LPR +T + +  + 
Sbjct  178  LNGITVRNKRLKVSYARPGGESIKDTNLYVTNLPRTITDDQLDTIF  223


>ref|NP_001259304.1| Sex lethal, isoform Y [Drosophila melanogaster]
 gb|AGB95149.1| Sex lethal, isoform Y [Drosophila melanogaster]
Length=708

 Score = 45.1 bits (105),  Expect = 0.30, Method: Compositional matrix adjust.
 Identities = 29/106 (27%), Positives = 49/106 (46%), Gaps = 10/106 (9%)

Query  119  NILIEHLPPQMTKRELKALF------NELDIRVNSIVKIQNGRAFVALNGEHSLQRILAL  172
            N+++ +LP  MT REL ALF      N   I  +       G AFV    E   QR + +
Sbjct  126  NLIVNYLPQDMTDRELYALFRAIGPINTCRIMRDYKTGYSFGYAFVDFTSEMDSQRAIKV  185

Query  173  DGNLTLMQKKVLVTQIGP----LKSSWRFTMDLPRDVTPEDIKKVL  214
               +T+  K++ V+   P    +K +  +  +LPR +T + +  + 
Sbjct  186  LNGITVRNKRLKVSYARPGGESIKDTNLYVTNLPRTITDDQLDTIF  231


>ref|NP_727160.2| Sex lethal, isoform J [Drosophila melanogaster]
 ref|NP_727166.2| Sex lethal, isoform G [Drosophila melanogaster]
 gb|AAN09198.1| Sex lethal, isoform J [Drosophila melanogaster]
 gb|AAN09200.2| Sex lethal, isoform G [Drosophila melanogaster]
Length=346

 Score = 44.3 bits (103),  Expect = 0.31, Method: Compositional matrix adjust.
 Identities = 29/106 (27%), Positives = 49/106 (46%), Gaps = 10/106 (9%)

Query  119  NILIEHLPPQMTKRELKALF------NELDIRVNSIVKIQNGRAFVALNGEHSLQRILAL  172
            N+++ +LP  MT REL ALF      N   I  +       G AFV    E   QR + +
Sbjct  118  NLIVNYLPQDMTDRELYALFRAIGPINTCRIMRDYKTGYSFGYAFVDFTSEMDSQRAIKV  177

Query  173  DGNLTLMQKKVLVTQIGP----LKSSWRFTMDLPRDVTPEDIKKVL  214
               +T+  K++ V+   P    +K +  +  +LPR +T + +  + 
Sbjct  178  LNGITVRNKRLKVSYARPGGESIKDTNLYVTNLPRTITDDQLDTIF  223


>gb|AAB81985.1| Sex-lethal protein [Musca domestica]
Length=318

 Score = 44.3 bits (103),  Expect = 0.31, Method: Compositional matrix adjust.
 Identities = 29/106 (27%), Positives = 50/106 (47%), Gaps = 10/106 (9%)

Query  119  NILIEHLPPQMTKRELKALF------NELDIRVNSIVKIQNGRAFVALNGEHSLQRILAL  172
            N+++ +LP  MT REL ALF      N   I  +       G AFV    E   Q  +  
Sbjct  96   NLIVNYLPQDMTDRELYALFRTCGPINTCRIMKDYKTGYSFGYAFVDFASEIDAQNAIKT  155

Query  173  DGNLTLMQKKVLVTQIGP----LKSSWRFTMDLPRDVTPEDIKKVL  214
               +T+  K++ V+   P    +K +  +  +LPR +T ++++K+ 
Sbjct  156  VNGITVRNKRLKVSYARPGGESIKDTNLYVTNLPRTITDDELEKIF  201


>ref|NP_727161.3| Sex lethal, isoform W [Drosophila melanogaster]
 gb|ADY17790.1| RE26226p [Drosophila melanogaster]
 gb|AAF46241.4| Sex lethal, isoform W [Drosophila melanogaster]
Length=344

 Score = 44.3 bits (103),  Expect = 0.31, Method: Compositional matrix adjust.
 Identities = 29/106 (27%), Positives = 49/106 (46%), Gaps = 10/106 (9%)

Query  119  NILIEHLPPQMTKRELKALF------NELDIRVNSIVKIQNGRAFVALNGEHSLQRILAL  172
            N+++ +LP  MT REL ALF      N   I  +       G AFV    E   QR + +
Sbjct  118  NLIVNYLPQDMTDRELYALFRAIGPINTCRIMRDYKTGYSFGYAFVDFTSEMDSQRAIKV  177

Query  173  DGNLTLMQKKVLVTQIGP----LKSSWRFTMDLPRDVTPEDIKKVL  214
               +T+  K++ V+   P    +K +  +  +LPR +T + +  + 
Sbjct  178  LNGITVRNKRLKVSYARPGGESIKDTNLYVTNLPRTITDDQLDTIF  223


>gb|ADX35950.1| SD08075p [Drosophila melanogaster]
Length=322

 Score = 44.3 bits (103),  Expect = 0.32, Method: Compositional matrix adjust.
 Identities = 33/125 (26%), Positives = 56/125 (45%), Gaps = 19/125 (15%)

Query  119  NILIEHLPPQMTKRELKALF------NELDIRVNSIVKIQNGRAFVALNGEHSLQRILAL  172
            N+++ +LP  MT REL ALF      N   I  +       G AFV    E   QR + +
Sbjct  94   NLIVNYLPQDMTDRELYALFRAIGPINTCRIMRDYKTGYSFGYAFVDFTSEMDSQRAIKV  153

Query  173  DGNLTLMQKKVLVTQIGP----LKSSWRFTMDLPRDVTPEDIKKVLQPLNVDILYNKKLP  228
               +T+  K++ V+   P    +K +  +  +LPR +T + +         D ++ K  P
Sbjct  154  LNGITVRNKRLKVSYARPGGESIKDTNLYVTNLPRTITDDQL---------DTIFGKYGP  204

Query  229  VGEPN  233
            + + N
Sbjct  205  IVQKN  209


>ref|XP_011296430.1| PREDICTED: sex-lethal homolog isoform X3 [Musca domestica]
Length=333

 Score = 44.3 bits (103),  Expect = 0.33, Method: Compositional matrix adjust.
 Identities = 29/106 (27%), Positives = 50/106 (47%), Gaps = 10/106 (9%)

Query  119  NILIEHLPPQMTKRELKALF------NELDIRVNSIVKIQNGRAFVALNGEHSLQRILAL  172
            N+++ +LP  MT REL ALF      N   I  +       G AFV    E   Q  +  
Sbjct  111  NLIVNYLPQDMTDRELYALFRTCGPINTCRIMKDYKTGYSFGYAFVDFASEIDAQNAIKT  170

Query  173  DGNLTLMQKKVLVTQIGP----LKSSWRFTMDLPRDVTPEDIKKVL  214
               +T+  K++ V+   P    +K +  +  +LPR +T ++++K+ 
Sbjct  171  VNGITVRNKRLKVSYARPGGESIKDTNLYVTNLPRTITDDELEKIF  216


>ref|NP_001027063.1| Sex lethal, isoform N [Drosophila melanogaster]
 ref|NP_524791.3| Sex lethal, isoform C [Drosophila melanogaster]
 gb|AAN09197.1| Sex lethal, isoform C [Drosophila melanogaster]
 gb|AAZ52510.1| Sex lethal, isoform N [Drosophila melanogaster]
 gb|ADV19045.1| RE22452p [Drosophila melanogaster]
Length=344

 Score = 44.3 bits (103),  Expect = 0.33, Method: Compositional matrix adjust.
 Identities = 29/106 (27%), Positives = 49/106 (46%), Gaps = 10/106 (9%)

Query  119  NILIEHLPPQMTKRELKALF------NELDIRVNSIVKIQNGRAFVALNGEHSLQRILAL  172
            N+++ +LP  MT REL ALF      N   I  +       G AFV    E   QR + +
Sbjct  116  NLIVNYLPQDMTDRELYALFRAIGPINTCRIMRDYKTGYSFGYAFVDFTSEMDSQRAIKV  175

Query  173  DGNLTLMQKKVLVTQIGP----LKSSWRFTMDLPRDVTPEDIKKVL  214
               +T+  K++ V+   P    +K +  +  +LPR +T + +  + 
Sbjct  176  LNGITVRNKRLKVSYARPGGESIKDTNLYVTNLPRTITDDQLDTIF  221


>ref|XP_011296433.1| PREDICTED: sex-lethal homolog isoform X7 [Musca domestica]
Length=319

 Score = 44.3 bits (103),  Expect = 0.33, Method: Compositional matrix adjust.
 Identities = 29/106 (27%), Positives = 50/106 (47%), Gaps = 10/106 (9%)

Query  119  NILIEHLPPQMTKRELKALF------NELDIRVNSIVKIQNGRAFVALNGEHSLQRILAL  172
            N+++ +LP  MT REL ALF      N   I  +       G AFV    E   Q  +  
Sbjct  83   NLIVNYLPQDMTDRELYALFRTCGPINTCRIMKDYKTGYSFGYAFVDFASEIDAQNAIKT  142

Query  173  DGNLTLMQKKVLVTQIGP----LKSSWRFTMDLPRDVTPEDIKKVL  214
               +T+  K++ V+   P    +K +  +  +LPR +T ++++K+ 
Sbjct  143  VNGITVRNKRLKVSYARPGGESIKDTNLYVTNLPRTITDDELEKIF  188


>ref|NP_001259303.1| Sex lethal, isoform X [Drosophila melanogaster]
 gb|AGB95148.1| Sex lethal, isoform X [Drosophila melanogaster]
Length=722

 Score = 44.7 bits (104),  Expect = 0.33, Method: Compositional matrix adjust.
 Identities = 29/106 (27%), Positives = 49/106 (46%), Gaps = 10/106 (9%)

Query  119  NILIEHLPPQMTKRELKALF------NELDIRVNSIVKIQNGRAFVALNGEHSLQRILAL  172
            N+++ +LP  MT REL ALF      N   I  +       G AFV    E   QR + +
Sbjct  118  NLIVNYLPQDMTDRELYALFRAIGPINTCRIMRDYKTGYSFGYAFVDFTSEMDSQRAIKV  177

Query  173  DGNLTLMQKKVLVTQIGP----LKSSWRFTMDLPRDVTPEDIKKVL  214
               +T+  K++ V+   P    +K +  +  +LPR +T + +  + 
Sbjct  178  LNGITVRNKRLKVSYARPGGESIKDTNLYVTNLPRTITDDQLDTIF  223


>ref|NP_001162688.1| Sex lethal, isoform R [Drosophila melanogaster]
 gb|ACZ95224.1| Sex lethal, isoform R [Drosophila melanogaster]
Length=342

 Score = 44.3 bits (103),  Expect = 0.33, Method: Compositional matrix adjust.
 Identities = 29/106 (27%), Positives = 49/106 (46%), Gaps = 10/106 (9%)

Query  119  NILIEHLPPQMTKRELKALF------NELDIRVNSIVKIQNGRAFVALNGEHSLQRILAL  172
            N+++ +LP  MT REL ALF      N   I  +       G AFV    E   QR + +
Sbjct  116  NLIVNYLPQDMTDRELYALFRAIGPINTCRIMRDYKTGYSFGYAFVDFTSEMDSQRAIKV  175

Query  173  DGNLTLMQKKVLVTQIGP----LKSSWRFTMDLPRDVTPEDIKKVL  214
               +T+  K++ V+   P    +K +  +  +LPR +T + +  + 
Sbjct  176  LNGITVRNKRLKVSYARPGGESIKDTNLYVTNLPRTITDDQLDTIF  221


>ref|NP_001162689.1| Sex lethal, isoform AB [Drosophila melanogaster]
 gb|ACZ95225.1| Sex lethal, isoform AB [Drosophila melanogaster]
Length=322

 Score = 44.3 bits (103),  Expect = 0.34, Method: Compositional matrix adjust.
 Identities = 29/106 (27%), Positives = 49/106 (46%), Gaps = 10/106 (9%)

Query  119  NILIEHLPPQMTKRELKALF------NELDIRVNSIVKIQNGRAFVALNGEHSLQRILAL  172
            N+++ +LP  MT REL ALF      N   I  +       G AFV    E   QR + +
Sbjct  94   NLIVNYLPQDMTDRELYALFRAIGPINTCRIMRDYKTGYSFGYAFVDFTSEMDSQRAIKV  153

Query  173  DGNLTLMQKKVLVTQIGP----LKSSWRFTMDLPRDVTPEDIKKVL  214
               +T+  K++ V+   P    +K +  +  +LPR +T + +  + 
Sbjct  154  LNGITVRNKRLKVSYARPGGESIKDTNLYVTNLPRTITDDQLDTIF  199


>ref|XP_002106359.1| Sex lethal [Drosophila simulans]
 gb|EDX17311.1| Sex lethal [Drosophila simulans]
Length=301

 Score = 43.9 bits (102),  Expect = 0.35, Method: Compositional matrix adjust.
 Identities = 29/106 (27%), Positives = 49/106 (46%), Gaps = 10/106 (9%)

Query  119  NILIEHLPPQMTKRELKALF------NELDIRVNSIVKIQNGRAFVALNGEHSLQRILAL  172
            N+++ +LP  MT REL ALF      N   I  +       G AFV    E   QR + +
Sbjct  118  NLIVNYLPQDMTDRELYALFRAIGPINTCRIMRDYKTGYSFGYAFVDFTSEMDSQRAIKV  177

Query  173  DGNLTLMQKKVLVTQIGP----LKSSWRFTMDLPRDVTPEDIKKVL  214
               +T+  K++ V+   P    +K +  +  +LPR +T + +  + 
Sbjct  178  LNGITVRNKRLKVSYARPGGESIKDTNLYVTNLPRTITDDQLDTIF  223


>ref|XP_011296429.1| PREDICTED: sex-lethal homolog isoform X2 [Musca domestica]
Length=339

 Score = 44.3 bits (103),  Expect = 0.35, Method: Compositional matrix adjust.
 Identities = 29/106 (27%), Positives = 50/106 (47%), Gaps = 10/106 (9%)

Query  119  NILIEHLPPQMTKRELKALF------NELDIRVNSIVKIQNGRAFVALNGEHSLQRILAL  172
            N+++ +LP  MT REL ALF      N   I  +       G AFV    E   Q  +  
Sbjct  103  NLIVNYLPQDMTDRELYALFRTCGPINTCRIMKDYKTGYSFGYAFVDFASEIDAQNAIKT  162

Query  173  DGNLTLMQKKVLVTQIGP----LKSSWRFTMDLPRDVTPEDIKKVL  214
               +T+  K++ V+   P    +K +  +  +LPR +T ++++K+ 
Sbjct  163  VNGITVRNKRLKVSYARPGGESIKDTNLYVTNLPRTITDDELEKIF  208


>ref|NP_727164.2| Sex lethal, isoform H [Drosophila melanogaster]
 gb|AAF46240.2| Sex lethal, isoform H [Drosophila melanogaster]
Length=366

 Score = 44.3 bits (103),  Expect = 0.35, Method: Compositional matrix adjust.
 Identities = 29/106 (27%), Positives = 49/106 (46%), Gaps = 10/106 (9%)

Query  119  NILIEHLPPQMTKRELKALF------NELDIRVNSIVKIQNGRAFVALNGEHSLQRILAL  172
            N+++ +LP  MT REL ALF      N   I  +       G AFV    E   QR + +
Sbjct  118  NLIVNYLPQDMTDRELYALFRAIGPINTCRIMRDYKTGYSFGYAFVDFTSEMDSQRAIKV  177

Query  173  DGNLTLMQKKVLVTQIGP----LKSSWRFTMDLPRDVTPEDIKKVL  214
               +T+  K++ V+   P    +K +  +  +LPR +T + +  + 
Sbjct  178  LNGITVRNKRLKVSYARPGGESIKDTNLYVTNLPRTITDDQLDTIF  223


>ref|XP_005177313.1| PREDICTED: sex-lethal homolog isoform X6 [Musca domestica]
Length=325

 Score = 43.9 bits (102),  Expect = 0.35, Method: Compositional matrix adjust.
 Identities = 29/106 (27%), Positives = 50/106 (47%), Gaps = 10/106 (9%)

Query  119  NILIEHLPPQMTKRELKALF------NELDIRVNSIVKIQNGRAFVALNGEHSLQRILAL  172
            N+++ +LP  MT REL ALF      N   I  +       G AFV    E   Q  +  
Sbjct  103  NLIVNYLPQDMTDRELYALFRTCGPINTCRIMKDYKTGYSFGYAFVDFASEIDAQNAIKT  162

Query  173  DGNLTLMQKKVLVTQIGP----LKSSWRFTMDLPRDVTPEDIKKVL  214
               +T+  K++ V+   P    +K +  +  +LPR +T ++++K+ 
Sbjct  163  VNGITVRNKRLKVSYARPGGESIKDTNLYVTNLPRTITDDELEKIF  208


>gb|ACY56908.1| RH48596p [Drosophila melanogaster]
Length=339

 Score = 44.3 bits (103),  Expect = 0.35, Method: Compositional matrix adjust.
 Identities = 29/106 (27%), Positives = 49/106 (46%), Gaps = 10/106 (9%)

Query  119  NILIEHLPPQMTKRELKALF------NELDIRVNSIVKIQNGRAFVALNGEHSLQRILAL  172
            N+++ +LP  MT REL ALF      N   I  +       G AFV    E   QR + +
Sbjct  111  NLIVNYLPQDMTDRELYALFRAIGPINTCRIMRDYKTGYSFGYAFVDFTSEMDSQRAIKV  170

Query  173  DGNLTLMQKKVLVTQIGP----LKSSWRFTMDLPRDVTPEDIKKVL  214
               +T+  K++ V+   P    +K +  +  +LPR +T + +  + 
Sbjct  171  LNGITVRNKRLKVSYARPGGESIKDTNLYVTNLPRTITDDQLDTIF  216


>pir||B39725 sex-lethal sex determination protein MS11 - fruit fly  (Drosophila 
melanogaster)
 gb|AAA28921.1| putative [Drosophila melanogaster]
Length=366

 Score = 44.3 bits (103),  Expect = 0.36, Method: Compositional matrix adjust.
 Identities = 29/106 (27%), Positives = 49/106 (46%), Gaps = 10/106 (9%)

Query  119  NILIEHLPPQMTKRELKALF------NELDIRVNSIVKIQNGRAFVALNGEHSLQRILAL  172
            N+++ +LP  MT REL ALF      N   I  +       G AFV    E   QR + +
Sbjct  118  NLIVNYLPQDMTDRELYALFRAIGPINTCRIMRDYKTGYSFGYAFVDFTSEMDSQRAIKV  177

Query  173  DGNLTLMQKKVLVTQIGP----LKSSWRFTMDLPRDVTPEDIKKVL  214
               +T+  K++ V+   P    +K +  +  +LPR +T + +  + 
Sbjct  178  LNGITVRNKRLKVSYARPGGESIKDTNLYVTNLPRTITDDQLDTIF  223


>gb|ADD20766.1| RH41387p [Drosophila melanogaster]
Length=322

 Score = 43.9 bits (102),  Expect = 0.36, Method: Compositional matrix adjust.
 Identities = 29/106 (27%), Positives = 49/106 (46%), Gaps = 10/106 (9%)

Query  119  NILIEHLPPQMTKRELKALF------NELDIRVNSIVKIQNGRAFVALNGEHSLQRILAL  172
            N+++ +LP  MT REL ALF      N   I  +       G AFV    E   QR + +
Sbjct  94   NLIVNYLPQDMTDRELYALFRAIGPINTCRIMRDYKTGYSFGYAFVDFTSEMDSQRAIKV  153

Query  173  DGNLTLMQKKVLVTQIGP----LKSSWRFTMDLPRDVTPEDIKKVL  214
               +T+  K++ V+   P    +K +  +  +LPR +T + +  + 
Sbjct  154  LNGITVRNKRLKVSYARPGGESIKDTNLYVTNLPRTITDDQLDTIF  199


>sp|Q24668|SXL_DROSU RecName: Full=Protein sex-lethal [Drosophila subobscura]
Length=354

 Score = 44.3 bits (103),  Expect = 0.36, Method: Compositional matrix adjust.
 Identities = 29/106 (27%), Positives = 49/106 (46%), Gaps = 10/106 (9%)

Query  119  NILIEHLPPQMTKRELKALF------NELDIRVNSIVKIQNGRAFVALNGEHSLQRILAL  172
            N+++ +LP  MT REL ALF      N   I  +       G AFV    E   QR + +
Sbjct  128  NLIVNYLPQDMTDRELYALFRAIGPINTCRIMRDYKTGYSFGYAFVDFTSEMDSQRAIKV  187

Query  173  DGNLTLMQKKVLVTQIGP----LKSSWRFTMDLPRDVTPEDIKKVL  214
               +T+  K++ V+   P    +K +  +  +LPR +T + +  + 
Sbjct  188  LNGITVRNKRLKVSYARPGGESIKDTNLYVTNLPRTITDDQLDTIF  233


>ref|NP_001027062.1| Sex lethal, isoform O [Drosophila melanogaster]
 gb|AAZ52509.1| Sex lethal, isoform O [Drosophila melanogaster]
Length=364

 Score = 44.3 bits (103),  Expect = 0.37, Method: Compositional matrix adjust.
 Identities = 29/106 (27%), Positives = 49/106 (46%), Gaps = 10/106 (9%)

Query  119  NILIEHLPPQMTKRELKALF------NELDIRVNSIVKIQNGRAFVALNGEHSLQRILAL  172
            N+++ +LP  MT REL ALF      N   I  +       G AFV    E   QR + +
Sbjct  116  NLIVNYLPQDMTDRELYALFRAIGPINTCRIMRDYKTGYSFGYAFVDFTSEMDSQRAIKV  175

Query  173  DGNLTLMQKKVLVTQIGP----LKSSWRFTMDLPRDVTPEDIKKVL  214
               +T+  K++ V+   P    +K +  +  +LPR +T + +  + 
Sbjct  176  LNGITVRNKRLKVSYARPGGESIKDTNLYVTNLPRTITDDQLDTIF  221


>pdb|4QQB|A Chain A, Structural Basis For The Assembly Of The Sxl-unr Translation 
Regulatory Complex
 pdb|4QQB|B Chain B, Structural Basis For The Assembly Of The Sxl-unr Translation 
Regulatory Complex
Length=176

 Score = 42.7 bits (99),  Expect = 0.39, Method: Compositional matrix adjust.
 Identities = 29/106 (27%), Positives = 49/106 (46%), Gaps = 10/106 (9%)

Query  119  NILIEHLPPQMTKRELKALF------NELDIRVNSIVKIQNGRAFVALNGEHSLQRILAL  172
            N+++ +LP  MT REL ALF      N   I  +       G AFV    E   QR + +
Sbjct  8    NLIVNYLPQDMTDRELYALFRAIGPINTCRIMRDYKTGYSFGYAFVDFTSEMDSQRAIKV  67

Query  173  DGNLTLMQKKVLVTQIGP----LKSSWRFTMDLPRDVTPEDIKKVL  214
               +T+  K++ V+   P    +K +  +  +LPR +T + +  + 
Sbjct  68   LNGITVRNKRLKVSYARPGGESIKDTNLYVTNLPRTITDDQLDTIF  113


>ref|NP_727163.2| Sex lethal, isoform E [Drosophila melanogaster]
 ref|NP_727165.2| Sex lethal, isoform A [Drosophila melanogaster]
 gb|AAO41638.2| Sex lethal, isoform A [Drosophila melanogaster]
 gb|AAZ52512.1| Sex lethal, isoform E [Drosophila melanogaster]
Length=314

 Score = 43.9 bits (102),  Expect = 0.41, Method: Compositional matrix adjust.
 Identities = 29/106 (27%), Positives = 49/106 (46%), Gaps = 10/106 (9%)

Query  119  NILIEHLPPQMTKRELKALF------NELDIRVNSIVKIQNGRAFVALNGEHSLQRILAL  172
            N+++ +LP  MT REL ALF      N   I  +       G AFV    E   QR + +
Sbjct  86   NLIVNYLPQDMTDRELYALFRAIGPINTCRIMRDYKTGYSFGYAFVDFTSEMDSQRAIKV  145

Query  173  DGNLTLMQKKVLVTQIGP----LKSSWRFTMDLPRDVTPEDIKKVL  214
               +T+  K++ V+   P    +K +  +  +LPR +T + +  + 
Sbjct  146  LNGITVRNKRLKVSYARPGGESIKDTNLYVTNLPRTITDDQLDTIF  191


>ref|XP_002025140.1| GL26885 [Drosophila persimilis]
 ref|XP_002133706.1| GA22653 [Drosophila pseudoobscura pseudoobscura]
 gb|EDW30628.1| GL26885 [Drosophila persimilis]
 gb|EDY72333.1| GA22653 [Drosophila pseudoobscura pseudoobscura]
Length=377

 Score = 43.9 bits (102),  Expect = 0.41, Method: Compositional matrix adjust.
 Identities = 29/106 (27%), Positives = 49/106 (46%), Gaps = 10/106 (9%)

Query  119  NILIEHLPPQMTKRELKALF------NELDIRVNSIVKIQNGRAFVALNGEHSLQRILAL  172
            N+++ +LP  MT REL ALF      N   I  +       G AFV    E   QR + +
Sbjct  128  NLIVNYLPQDMTDRELYALFRAIGPINTCRIMRDYKTGYSFGYAFVDFTSEMDSQRAIKV  187

Query  173  DGNLTLMQKKVLVTQIGP----LKSSWRFTMDLPRDVTPEDIKKVL  214
               +T+  K++ V+   P    +K +  +  +LPR +T + +  + 
Sbjct  188  LNGITVRNKRLKVSYARPGGESIKDTNLYVTNLPRTITDDQLDTIF  233


>ref|XP_011296431.1| PREDICTED: sex-lethal homolog isoform X4 [Musca domestica]
Length=332

 Score = 43.9 bits (102),  Expect = 0.41, Method: Compositional matrix adjust.
 Identities = 29/106 (27%), Positives = 50/106 (47%), Gaps = 10/106 (9%)

Query  119  NILIEHLPPQMTKRELKALF------NELDIRVNSIVKIQNGRAFVALNGEHSLQRILAL  172
            N+++ +LP  MT REL ALF      N   I  +       G AFV    E   Q  +  
Sbjct  111  NLIVNYLPQDMTDRELYALFRTCGPINTCRIMKDYKTGYSFGYAFVDFASEIDAQNAIKT  170

Query  173  DGNLTLMQKKVLVTQIGP----LKSSWRFTMDLPRDVTPEDIKKVL  214
               +T+  K++ V+   P    +K +  +  +LPR +T ++++K+ 
Sbjct  171  VNGITVRNKRLKVSYARPGGESIKDTNLYVTNLPRTITDDELEKIF  216


>emb|CAA67016.1| sex-lethal [Drosophila subobscura]
Length=346

 Score = 43.9 bits (102),  Expect = 0.42, Method: Compositional matrix adjust.
 Identities = 29/106 (27%), Positives = 49/106 (46%), Gaps = 10/106 (9%)

Query  119  NILIEHLPPQMTKRELKALF------NELDIRVNSIVKIQNGRAFVALNGEHSLQRILAL  172
            N+++ +LP  MT REL ALF      N   I  +       G AFV    E   QR + +
Sbjct  120  NLIVNYLPQDMTDRELYALFRAIGPINTCRIMRDYKTGYSFGYAFVDFTSEMDSQRAIKV  179

Query  173  DGNLTLMQKKVLVTQIGP----LKSSWRFTMDLPRDVTPEDIKKVL  214
               +T+  K++ V+   P    +K +  +  +LPR +T + +  + 
Sbjct  180  LNGITVRNKRLKVSYARPGGESIKDTNLYVTNLPRTITDDQLDTIF  225


>ref|XP_001978518.1| GG17637 [Drosophila erecta]
 ref|XP_002101294.1| GE17544 [Drosophila yakuba]
 gb|EDV47445.1| GG17637 [Drosophila erecta]
 gb|EDX02402.1| GE17544 [Drosophila yakuba]
Length=335

 Score = 43.5 bits (101),  Expect = 0.51, Method: Compositional matrix adjust.
 Identities = 29/106 (27%), Positives = 49/106 (46%), Gaps = 10/106 (9%)

Query  119  NILIEHLPPQMTKRELKALF------NELDIRVNSIVKIQNGRAFVALNGEHSLQRILAL  172
            N+++ +LP  MT REL ALF      N   I  +       G AFV    E   QR + +
Sbjct  86   NLIVNYLPQDMTDRELYALFRAIGPINTCRIMRDYKTGYSFGYAFVDFTSEMDSQRAIKV  145

Query  173  DGNLTLMQKKVLVTQIGP----LKSSWRFTMDLPRDVTPEDIKKVL  214
               +T+  K++ V+   P    +K +  +  +LPR +T + +  + 
Sbjct  146  LNGITVRNKRLKVSYARPGGESIKDTNLYVTNLPRTITDDQLDTIF  191


>ref|NP_001274464.1| sex-lethal homolog [Musca domestica]
 sp|O17310|SXL_MUSDO RecName: Full=Sex-lethal homolog [Musca domestica]
 gb|AAB81986.1| Sex-lethal protein [Musca domestica]
Length=324

 Score = 43.5 bits (101),  Expect = 0.54, Method: Compositional matrix adjust.
 Identities = 29/106 (27%), Positives = 50/106 (47%), Gaps = 10/106 (9%)

Query  119  NILIEHLPPQMTKRELKALF------NELDIRVNSIVKIQNGRAFVALNGEHSLQRILAL  172
            N+++ +LP  MT REL ALF      N   I  +       G AFV    E   Q  +  
Sbjct  103  NLIVNYLPQDMTDRELYALFRTCGPINTCRIMKDYKTGYSFGYAFVDFASEIDAQNAIKT  162

Query  173  DGNLTLMQKKVLVTQIGP----LKSSWRFTMDLPRDVTPEDIKKVL  214
               +T+  K++ V+   P    +K +  +  +LPR +T ++++K+ 
Sbjct  163  VNGITVRNKRLKVSYARPGGESIKDTNLYVTNLPRTITDDELEKIF  208


>gb|AAK20026.1|AF234184_1 sex-lethal protein SXL2 [Lucilia cuprina]
Length=325

 Score = 43.1 bits (100),  Expect = 0.66, Method: Compositional matrix adjust.
 Identities = 29/106 (27%), Positives = 50/106 (47%), Gaps = 10/106 (9%)

Query  119  NILIEHLPPQMTKRELKALF------NELDIRVNSIVKIQNGRAFVALNGEHSLQRILAL  172
            N+++ +LP  MT REL ALF      N   I  +       G AFV    E   Q  +  
Sbjct  103  NLIVNYLPQDMTDRELYALFRTCGPINTCRIMKDYKTGYSFGYAFVDFASEIDAQNAIKS  162

Query  173  DGNLTLMQKKVLVTQIGP----LKSSWRFTMDLPRDVTPEDIKKVL  214
               +T+  K++ V+   P    +K +  +  +LPR +T ++++K+ 
Sbjct  163  LNGVTVRNKRLKVSYARPGGESIKDTNLYVTNLPRTITDDELEKIF  208


>gb|AAK20025.1|AF234183_1 sex-lethal protein SXL1 [Lucilia cuprina]
Length=324

 Score = 43.1 bits (100),  Expect = 0.70, Method: Compositional matrix adjust.
 Identities = 29/106 (27%), Positives = 50/106 (47%), Gaps = 10/106 (9%)

Query  119  NILIEHLPPQMTKRELKALF------NELDIRVNSIVKIQNGRAFVALNGEHSLQRILAL  172
            N+++ +LP  MT REL ALF      N   I  +       G AFV    E   Q  +  
Sbjct  103  NLIVNYLPQDMTDRELYALFRTCGPINTCRIMKDYKTGYSFGYAFVDFASEIDAQNAIKS  162

Query  173  DGNLTLMQKKVLVTQIGP----LKSSWRFTMDLPRDVTPEDIKKVL  214
               +T+  K++ V+   P    +K +  +  +LPR +T ++++K+ 
Sbjct  163  LNGVTVRNKRLKVSYARPGGESIKDTNLYVTNLPRTITDDELEKIF  208


>sp|O97018|SXL_CHRRU RecName: Full=Sex-lethal homolog [Chrysomya rufifacies]
 gb|AAN74807.1| sex-lethal [Chrysomya rufifacies]
Length=307

 Score = 43.1 bits (100),  Expect = 0.70, Method: Compositional matrix adjust.
 Identities = 29/106 (27%), Positives = 50/106 (47%), Gaps = 10/106 (9%)

Query  119  NILIEHLPPQMTKRELKALF------NELDIRVNSIVKIQNGRAFVALNGEHSLQRILAL  172
            N+++ +LP  MT REL ALF      N   I  +       G AFV    E   Q  +  
Sbjct  86   NLIVNYLPQDMTDRELYALFRTCGPINTCRIMKDYKTGYSFGYAFVDFASEIDAQNAIKS  145

Query  173  DGNLTLMQKKVLVTQIGP----LKSSWRFTMDLPRDVTPEDIKKVL  214
               +T+  K++ V+   P    +K +  +  +LPR +T ++++K+ 
Sbjct  146  LNGVTVRNKRLKVSYARPGGESIKDTNLYVTNLPRTITDDELEKIF  191


>emb|CDS18761.1| ELAV protein 2 [Echinococcus granulosus]
Length=498

 Score = 43.5 bits (101),  Expect = 0.73, Method: Compositional matrix adjust.
 Identities = 35/139 (25%), Positives = 67/139 (48%), Gaps = 18/139 (13%)

Query  93   KSNDKHIASAPLNTNNV------SEEKARELCNILIEHLPPQMTKRELKALFNELDIRVN  146
            KSN+K   S+ L+          +E  +    N+++ +LP  MT+ E++ALFN +  +V+
Sbjct  13   KSNEKSQRSSSLDVETSPSAQTKAETASENKTNLIVNYLPQSMTQEEIRALFNTIG-KVS  71

Query  147  SIVKIQN-------GRAFVALNGEHSLQRILALDGNLTLMQKKVLVTQIGP----LKSSW  195
            S   I++       G  FV        ++ +     + L  KK+ V+   P    +K + 
Sbjct  72   SCKLIRDKSTGQSLGYGFVNYAYASDAEKAIKHFNGMQLKNKKIKVSIARPSSESIKGAN  131

Query  196  RFTMDLPRDVTPEDIKKVL  214
             +   LP++V+ ED++K+ 
Sbjct  132  LYICGLPKNVSQEDLEKIF  150


>emb|CDJ01832.1| ELAV protein 2 [Echinococcus multilocularis]
 emb|CDS39114.1| ELAV protein 2 [Echinococcus multilocularis]
Length=498

 Score = 43.1 bits (100),  Expect = 0.81, Method: Compositional matrix adjust.
 Identities = 29/118 (25%), Positives = 59/118 (50%), Gaps = 12/118 (10%)

Query  108  NVSEEKARELCNILIEHLPPQMTKRELKALFNELDIRVNSIVKIQN-------GRAFVAL  160
            N +E  +    N+++ +LP  MT+ E+++LFN +  +V+S   I++       G  FV  
Sbjct  34   NKAETASENKTNLIVNYLPQSMTQEEIRSLFNSIG-KVSSCKLIRDKSTGQSLGYGFVNY  92

Query  161  NGEHSLQRILALDGNLTLMQKKVLVTQIGP----LKSSWRFTMDLPRDVTPEDIKKVL  214
                  ++ +     + L  KK+ V+   P    +K +  +   LP++V+ ED++K+ 
Sbjct  93   AYASDAEKAIKHFNGMQLKNKKIKVSIARPSSESIKGANLYICGLPKNVSQEDLEKIF  150


>gb|EUB55408.1| ELAV-like protein [Echinococcus granulosus]
Length=483

 Score = 43.1 bits (100),  Expect = 0.91, Method: Compositional matrix adjust.
 Identities = 35/139 (25%), Positives = 67/139 (48%), Gaps = 18/139 (13%)

Query  93   KSNDKHIASAPLNTNNV------SEEKARELCNILIEHLPPQMTKRELKALFNELDIRVN  146
            KSN+K   S+ L+          +E  +    N+++ +LP  MT+ E++ALFN +  +V+
Sbjct  13   KSNEKSQRSSSLDVETSPSAQTKAETASENKTNLIVNYLPQSMTQEEIRALFNTIG-KVS  71

Query  147  SIVKIQN-------GRAFVALNGEHSLQRILALDGNLTLMQKKVLVTQIGP----LKSSW  195
            S   I++       G  FV        ++ +     + L  KK+ V+   P    +K + 
Sbjct  72   SCKLIRDKSTGQSLGYGFVNYAYASDAEKAIKHFNGMQLKNKKIKVSIARPSSESIKGAN  131

Query  196  RFTMDLPRDVTPEDIKKVL  214
             +   LP++V+ ED++K+ 
Sbjct  132  LYICGLPKNVSQEDLEKIF  150


>gb|AAS45603.1| sex-lethal [Bradysia coprophila]
Length=289

 Score = 42.4 bits (98),  Expect = 0.99, Method: Compositional matrix adjust.
 Identities = 36/139 (26%), Positives = 63/139 (45%), Gaps = 16/139 (12%)

Query  119  NILIEHLPPQMTKRELKALFNELD-IRVNSIVK-----IQNGRAFVALNGEHSLQRILAL  172
            N+++ +LP  MT RE  ALF+    I  + I++        G  FV    E   QR +  
Sbjct  76   NLIVNYLPQDMTDREFYALFSTTGPIESSRIMRDFKTCYSFGYGFVNYLTEEGAQRAIKS  135

Query  173  DGNLTLMQKKVLVTQIGP----LKSSWRFTMDLPRDVTPEDIKKVLQPLNV----DILYN  224
               +T+  K++ V+   P    LK +  +  +LPR +T E + ++     +    +IL +
Sbjct  136  LNGVTVRNKRLKVSYARPAGEELKETNLYVTNLPRTITEEQLDEIFGKYGLIVQKNILRD  195

Query  225  KKLPVGEPNGDKRVLQNDK  243
            K    G+P G   +  N +
Sbjct  196  KM--TGKPRGVAFIRYNKR  212


>sp|O61374|SXL_CERCA RecName: Full=Sex-lethal homolog; AltName: Full=CCSXL [Ceratitis 
capitata]
Length=348

 Score = 42.7 bits (99),  Expect = 1.1, Method: Compositional matrix adjust.
 Identities = 35/137 (26%), Positives = 59/137 (43%), Gaps = 12/137 (9%)

Query  119  NILIEHLPPQMTKRELKALF------NELDIRVNSIVKIQNGRAFVALNGEHSLQRILAL  172
            N+++ +LP  MT REL ALF      N   I  +       G AFV    E   QR +  
Sbjct  111  NLIVNYLPQDMTDRELYALFRTIGPINTCRIMRDYKTGYSFGYAFVDFAAETDSQRAIKS  170

Query  173  DGNLTLMQKKVLVTQIGP----LKSSWRFTMDLPRDVTPEDIKKVLQPLNVDILYN--KK  226
               +T+  K++ V+   P    +K +  +  +LPR +T + +  +     + +  N  + 
Sbjct  171  LNGITVRNKRLKVSYARPGGESIKDTNLYVTNLPRTITDDQLDTIFGKYGMIVQKNILRD  230

Query  227  LPVGEPNGDKRVLQNDK  243
               G+P G   V  N +
Sbjct  231  KLTGKPRGVAFVRFNKR  247


>ref|XP_012158510.1| PREDICTED: sex-lethal homolog isoform X4 [Ceratitis capitata]
Length=329

 Score = 42.4 bits (98),  Expect = 1.2, Method: Compositional matrix adjust.
 Identities = 35/137 (26%), Positives = 59/137 (43%), Gaps = 12/137 (9%)

Query  119  NILIEHLPPQMTKRELKALF------NELDIRVNSIVKIQNGRAFVALNGEHSLQRILAL  172
            N+++ +LP  MT REL ALF      N   I  +       G AFV    E   QR +  
Sbjct  112  NLIVNYLPQDMTDRELYALFRTIGPINTCRIMRDYKTGYSFGYAFVDFAAETDSQRAIKS  171

Query  173  DGNLTLMQKKVLVTQIGP----LKSSWRFTMDLPRDVTPEDIKKVLQPLNVDILYN--KK  226
               +T+  K++ V+   P    +K +  +  +LPR +T + +  +     + +  N  + 
Sbjct  172  LNGITVRNKRLKVSYARPGGESIKDTNLYVTNLPRTITDDQLDTIFGKYGMIVQKNILRD  231

Query  227  LPVGEPNGDKRVLQNDK  243
               G+P G   V  N +
Sbjct  232  KLTGKPRGVAFVRFNKR  248


>ref|XP_008414224.1| PREDICTED: ribonucleoprotein PTB-binding 1 isoform X2 [Poecilia 
reticulata]
Length=735

 Score = 43.1 bits (100),  Expect = 1.2, Method: Compositional matrix adjust.
 Identities = 46/186 (25%), Positives = 84/186 (45%), Gaps = 12/186 (6%)

Query  39   DDSARASPIFVNRPPPFDKNDYLLPSMVNKTEPIRNSRSSEASSRHPTSYTP--WNKSND  96
            D+ A +   FV+RP  F  N  L     N     R   +  AS     +  P    + ++
Sbjct  14   DEGADSGATFVSRP--FSANHDLATDKENFMAGDRQRYTEFASVEEDATSGPECQRRVDE  71

Query  97   KHIASAPLNTNNVSEEKARELCN---ILIEHLPPQMTKRELKALFNELDIRVNSIVKIQN  153
              I  +P +  +  E   RE  N   I+I++LPP +T +E+  L    D++   + K + 
Sbjct  72   DLIPLSPEDIESRLERTRREFYNRRKIIIKNLPPDVTNQEVHDLLGNYDLKYCFVDKYK-  130

Query  154  GRAFVA-LNGEHSLQRILALDGNLTLMQKKVLVTQIGPLKSSWRFTMDLPRDVTPEDIKK  212
            G AFV  LNGE +   I     +  +++++ +  Q+ P   +     +LPR  T +  ++
Sbjct  131  GTAFVTLLNGEQAQCAIKEF--HQIMLREREISVQLQP-TDALLCIANLPRAFTQQQFEE  187

Query  213  VLQPLN  218
            +++P  
Sbjct  188  LVRPFG  193


>ref|XP_012158507.1| PREDICTED: sex-lethal homolog isoform X1 [Ceratitis capitata]
Length=350

 Score = 42.4 bits (98),  Expect = 1.2, Method: Compositional matrix adjust.
 Identities = 29/106 (27%), Positives = 48/106 (45%), Gaps = 10/106 (9%)

Query  119  NILIEHLPPQMTKRELKALF------NELDIRVNSIVKIQNGRAFVALNGEHSLQRILAL  172
            N+++ +LP  MT REL ALF      N   I  +       G AFV    E   QR +  
Sbjct  112  NLIVNYLPQDMTDRELYALFRTIGPINTCRIMRDYKTGYSFGYAFVDFAAETDSQRAIKS  171

Query  173  DGNLTLMQKKVLVTQIGP----LKSSWRFTMDLPRDVTPEDIKKVL  214
               +T+  K++ V+   P    +K +  +  +LPR +T + +  + 
Sbjct  172  LNGITVRNKRLKVSYARPGGESIKDTNLYVTNLPRTITDDQLDTIF  217


>ref|XP_011176864.1| PREDICTED: sex-lethal homolog isoform X4 [Bactrocera cucurbitae]
Length=354

 Score = 42.4 bits (98),  Expect = 1.2, Method: Compositional matrix adjust.
 Identities = 29/106 (27%), Positives = 48/106 (45%), Gaps = 10/106 (9%)

Query  119  NILIEHLPPQMTKRELKALF------NELDIRVNSIVKIQNGRAFVALNGEHSLQRILAL  172
            N+++ +LP  MT REL ALF      N   I  +       G AFV    E   QR +  
Sbjct  119  NLIVNYLPQDMTDRELYALFRTIGPINTCRIMRDYKTGYSFGYAFVDFASERDSQRAIKS  178

Query  173  DGNLTLMQKKVLVTQIGP----LKSSWRFTMDLPRDVTPEDIKKVL  214
               +T+  K++ V+   P    +K +  +  +LPR +T + +  + 
Sbjct  179  LNGITVRNKRLKVSYARPGGESIKDTNLYVTNLPRTITDDQLDTIF  224


>ref|XP_012158508.1| PREDICTED: sex-lethal homolog isoform X2 [Ceratitis capitata]
Length=349

 Score = 42.4 bits (98),  Expect = 1.3, Method: Compositional matrix adjust.
 Identities = 35/137 (26%), Positives = 59/137 (43%), Gaps = 12/137 (9%)

Query  119  NILIEHLPPQMTKRELKALF------NELDIRVNSIVKIQNGRAFVALNGEHSLQRILAL  172
            N+++ +LP  MT REL ALF      N   I  +       G AFV    E   QR +  
Sbjct  112  NLIVNYLPQDMTDRELYALFRTIGPINTCRIMRDYKTGYSFGYAFVDFAAETDSQRAIKS  171

Query  173  DGNLTLMQKKVLVTQIGP----LKSSWRFTMDLPRDVTPEDIKKVLQPLNVDILYN--KK  226
               +T+  K++ V+   P    +K +  +  +LPR +T + +  +     + +  N  + 
Sbjct  172  LNGITVRNKRLKVSYARPGGESIKDTNLYVTNLPRTITDDQLDTIFGKYGMIVQKNILRD  231

Query  227  LPVGEPNGDKRVLQNDK  243
               G+P G   V  N +
Sbjct  232  KLTGKPRGVAFVRFNKR  248


>pdb|3SXL|A Chain A, Sex-Lethal Rna Recognition Domains 1 And 2 From Drosophila 
Melanogaster
 pdb|3SXL|B Chain B, Sex-Lethal Rna Recognition Domains 1 And 2 From Drosophila 
Melanogaster
 pdb|3SXL|C Chain C, Sex-Lethal Rna Recognition Domains 1 And 2 From Drosophila 
Melanogaster
Length=184

 Score = 41.2 bits (95),  Expect = 1.3, Method: Compositional matrix adjust.
 Identities = 28/106 (26%), Positives = 48/106 (45%), Gaps = 10/106 (9%)

Query  119  NILIEHLPPQMTKRELKALF------NELDIRVNSIVKIQNGRAFVALNGEHSLQRILAL  172
            N+++ +LP   T REL ALF      N   I  +       G AFV    E   QR + +
Sbjct  16   NLIVNYLPQDXTDRELYALFRAIGPINTCRIXRDYKTGYSFGYAFVDFTSEXDSQRAIKV  75

Query  173  DGNLTLMQKKVLVTQIGP----LKSSWRFTMDLPRDVTPEDIKKVL  214
               +T+  K++ V+   P    +K +  +  +LPR +T + +  + 
Sbjct  76   LNGITVRNKRLKVSYARPGGESIKDTNLYVTNLPRTITDDQLDTIF  121


>ref|XP_008414223.1| PREDICTED: ribonucleoprotein PTB-binding 1 isoform X1 [Poecilia 
reticulata]
Length=747

 Score = 42.7 bits (99),  Expect = 1.3, Method: Compositional matrix adjust.
 Identities = 46/186 (25%), Positives = 84/186 (45%), Gaps = 12/186 (6%)

Query  39   DDSARASPIFVNRPPPFDKNDYLLPSMVNKTEPIRNSRSSEASSRHPTSYTP--WNKSND  96
            D+ A +   FV+RP  F  N  L     N     R   +  AS     +  P    + ++
Sbjct  14   DEGADSGATFVSRP--FSANHDLATDKENFMAGDRQRYTEFASVEEDATSGPECQRRVDE  71

Query  97   KHIASAPLNTNNVSEEKARELCN---ILIEHLPPQMTKRELKALFNELDIRVNSIVKIQN  153
              I  +P +  +  E   RE  N   I+I++LPP +T +E+  L    D++   + K + 
Sbjct  72   DLIPLSPEDIESRLERTRREFYNRRKIIIKNLPPDVTNQEVHDLLGNYDLKYCFVDKYK-  130

Query  154  GRAFVA-LNGEHSLQRILALDGNLTLMQKKVLVTQIGPLKSSWRFTMDLPRDVTPEDIKK  212
            G AFV  LNGE +   I     +  +++++ +  Q+ P   +     +LPR  T +  ++
Sbjct  131  GTAFVTLLNGEQAQCAIKEF--HQIMLREREISVQLQP-TDALLCIANLPRAFTQQQFEE  187

Query  213  VLQPLN  218
            +++P  
Sbjct  188  LVRPFG  193


>ref|XP_009903515.1| PREDICTED: ELAV-like protein 2 isoform X1 [Picoides pubescens]
Length=374

 Score = 42.4 bits (98),  Expect = 1.3, Method: Compositional matrix adjust.
 Identities = 37/143 (26%), Positives = 68/143 (48%), Gaps = 14/143 (10%)

Query  82   SRHPTSYTPWNKSNDKHIASAPLNTNNVSEEKARELCNILIEHLPPQMTKRELKALFNEL  141
            S  PT  +  + SN  +  S+P+++ N  + K     N+++ +LP  MT+ ELK+LF  +
Sbjct  35   SNGPTCNSTAHSSNTINNCSSPVDSGNTEDSKT----NLIVNYLPQNMTQEELKSLFGSI  90

Query  142  -DIRVNSIV--KIQN---GRAFVALNGEHSLQRILALDGNLTLMQKKVLVTQIGPLKSSW  195
             +I    +V  KI     G  FV        ++ +     L L  K + V+   P  +S 
Sbjct  91   GEIESCKLVRDKITGQSLGYGFVNYVDPKDAEKAINTLNGLRLQTKTIKVSYARPSSASI  150

Query  196  R----FTMDLPRDVTPEDIKKVL  214
            R    +   LP+ +T +D++++ 
Sbjct  151  RDANLYVSGLPKSMTQKDLEQLF  173


>ref|XP_012158509.1| PREDICTED: sex-lethal homolog isoform X3 [Ceratitis capitata]
Length=342

 Score = 42.4 bits (98),  Expect = 1.4, Method: Compositional matrix adjust.
 Identities = 29/106 (27%), Positives = 48/106 (45%), Gaps = 10/106 (9%)

Query  119  NILIEHLPPQMTKRELKALF------NELDIRVNSIVKIQNGRAFVALNGEHSLQRILAL  172
            N+++ +LP  MT REL ALF      N   I  +       G AFV    E   QR +  
Sbjct  104  NLIVNYLPQDMTDRELYALFRTIGPINTCRIMRDYKTGYSFGYAFVDFAAETDSQRAIKS  163

Query  173  DGNLTLMQKKVLVTQIGP----LKSSWRFTMDLPRDVTPEDIKKVL  214
               +T+  K++ V+   P    +K +  +  +LPR +T + +  + 
Sbjct  164  LNGITVRNKRLKVSYARPGGESIKDTNLYVTNLPRTITDDQLDTIF  209


>gb|KNC25619.1| hypothetical protein FF38_03391 [Lucilia cuprina]
Length=386

 Score = 42.4 bits (98),  Expect = 1.4, Method: Compositional matrix adjust.
 Identities = 29/106 (27%), Positives = 50/106 (47%), Gaps = 10/106 (9%)

Query  119  NILIEHLPPQMTKRELKALF------NELDIRVNSIVKIQNGRAFVALNGEHSLQRILAL  172
            N+++ +LP  MT REL ALF      N   I  +       G AFV    E   Q  +  
Sbjct  111  NLIVNYLPQDMTDRELYALFRTCGPINTCRIMKDYKTGYSFGYAFVDFASEIDAQNAIKS  170

Query  173  DGNLTLMQKKVLVTQIGP----LKSSWRFTMDLPRDVTPEDIKKVL  214
               +T+  K++ V+   P    +K +  +  +LPR +T ++++K+ 
Sbjct  171  LNGVTVRNKRLKVSYARPGGESIKDTNLYVTNLPRTITDDELEKIF  216


>gb|AIK25413.1| sex-lethal-like protein, partial [Bactrocera tryoni]
Length=338

 Score = 42.4 bits (98),  Expect = 1.4, Method: Compositional matrix adjust.
 Identities = 29/106 (27%), Positives = 48/106 (45%), Gaps = 10/106 (9%)

Query  119  NILIEHLPPQMTKRELKALF------NELDIRVNSIVKIQNGRAFVALNGEHSLQRILAL  172
            N+++ +LP  MT REL ALF      N   I  +       G AFV    E   QR +  
Sbjct  109  NLIVNYLPQDMTDRELYALFRTIGPINTCRIMRDYKTGYSFGYAFVDFASETGSQRAIKS  168

Query  173  DGNLTLMQKKVLVTQIGP----LKSSWRFTMDLPRDVTPEDIKKVL  214
               +T+  K++ V+   P    +K +  +  +LPR +T + +  + 
Sbjct  169  LNGITVRNKRLKVSYARPGGESIKDTNLYVTNLPRTITDDQLDTIF  214


>ref|XP_011176866.1| PREDICTED: sex-lethal homolog isoform X6 [Bactrocera cucurbitae]
Length=346

 Score = 42.4 bits (98),  Expect = 1.4, Method: Compositional matrix adjust.
 Identities = 29/106 (27%), Positives = 48/106 (45%), Gaps = 10/106 (9%)

Query  119  NILIEHLPPQMTKRELKALF------NELDIRVNSIVKIQNGRAFVALNGEHSLQRILAL  172
            N+++ +LP  MT REL ALF      N   I  +       G AFV    E   QR +  
Sbjct  111  NLIVNYLPQDMTDRELYALFRTIGPINTCRIMRDYKTGYSFGYAFVDFASERDSQRAIKS  170

Query  173  DGNLTLMQKKVLVTQIGP----LKSSWRFTMDLPRDVTPEDIKKVL  214
               +T+  K++ V+   P    +K +  +  +LPR +T + +  + 
Sbjct  171  LNGITVRNKRLKVSYARPGGESIKDTNLYVTNLPRTITDDQLDTIF  216


>ref|XP_009903516.1| PREDICTED: ELAV-like protein 2 isoform X2 [Picoides pubescens]
Length=387

 Score = 42.4 bits (98),  Expect = 1.5, Method: Compositional matrix adjust.
 Identities = 37/143 (26%), Positives = 68/143 (48%), Gaps = 14/143 (10%)

Query  82   SRHPTSYTPWNKSNDKHIASAPLNTNNVSEEKARELCNILIEHLPPQMTKRELKALFNEL  141
            S  PT  +  + SN  +  S+P+++ N  + K     N+++ +LP  MT+ ELK+LF  +
Sbjct  35   SNGPTCNSTAHSSNTINNCSSPVDSGNTEDSKT----NLIVNYLPQNMTQEELKSLFGSI  90

Query  142  -DIRVNSIV--KIQN---GRAFVALNGEHSLQRILALDGNLTLMQKKVLVTQIGPLKSSW  195
             +I    +V  KI     G  FV        ++ +     L L  K + V+   P  +S 
Sbjct  91   GEIESCKLVRDKITGQSLGYGFVNYVDPKDAEKAINTLNGLRLQTKTIKVSYARPSSASI  150

Query  196  R----FTMDLPRDVTPEDIKKVL  214
            R    +   LP+ +T +D++++ 
Sbjct  151  RDANLYVSGLPKSMTQKDLEQLF  173


>ref|XP_009685071.1| PREDICTED: ELAV-like protein 2 isoform X3 [Struthio camelus australis]
Length=359

 Score = 42.0 bits (97),  Expect = 1.6, Method: Compositional matrix adjust.
 Identities = 37/143 (26%), Positives = 67/143 (47%), Gaps = 14/143 (10%)

Query  82   SRHPTSYTPWNKSNDKHIASAPLNTNNVSEEKARELCNILIEHLPPQMTKRELKALFNEL  141
            S  PT     N SN  +  S+P+++ N  + K     N+++ +LP  MT+ ELK+LF  +
Sbjct  6    SNGPTCNNTANGSNAINNCSSPVDSGNTEDSKT----NLIVNYLPQNMTQEELKSLFGSI  61

Query  142  -DIRVNSIV--KIQN---GRAFVALNGEHSLQRILALDGNLTLMQKKVLVTQIGPLKSSW  195
             +I    +V  KI     G  FV        ++ +     L L  K + V+   P  +S 
Sbjct  62   GEIESCKLVRDKITGQSLGYGFVNYVDPKDAEKAINTLNGLRLQTKTIKVSYARPSSASI  121

Query  196  R----FTMDLPRDVTPEDIKKVL  214
            R    +   LP+ +T ++++++ 
Sbjct  122  RDANLYVSGLPKTMTQKELEQLF  144


>ref|NP_001266336.1| sex-lethal homolog [Ceratitis capitata]
 gb|AAC38968.1| sex-lethal homolog CcSXL [Ceratitis capitata]
Length=340

 Score = 42.0 bits (97),  Expect = 1.6, Method: Compositional matrix adjust.
 Identities = 29/106 (27%), Positives = 48/106 (45%), Gaps = 10/106 (9%)

Query  119  NILIEHLPPQMTKRELKALF------NELDIRVNSIVKIQNGRAFVALNGEHSLQRILAL  172
            N+++ +LP  MT REL ALF      N   I  +       G AFV    E   QR +  
Sbjct  103  NLIVNYLPQDMTDRELYALFRTIGPINTCRIMRDYKTGYSFGYAFVDFAAETDSQRAIKS  162

Query  173  DGNLTLMQKKVLVTQIGP----LKSSWRFTMDLPRDVTPEDIKKVL  214
               +T+  K++ V+   P    +K +  +  +LPR +T + +  + 
Sbjct  163  LNGITVRNKRLKVSYARPGGESIKDTNLYVTNLPRTITDDQLDTIF  208


>ref|XP_011176865.1| PREDICTED: sex-lethal homolog isoform X5 [Bactrocera cucurbitae]
Length=345

 Score = 42.0 bits (97),  Expect = 1.6, Method: Compositional matrix adjust.
 Identities = 29/106 (27%), Positives = 48/106 (45%), Gaps = 10/106 (9%)

Query  119  NILIEHLPPQMTKRELKALF------NELDIRVNSIVKIQNGRAFVALNGEHSLQRILAL  172
            N+++ +LP  MT REL ALF      N   I  +       G AFV    E   QR +  
Sbjct  111  NLIVNYLPQDMTDRELYALFRTIGPINTCRIMRDYKTGYSFGYAFVDFASERDSQRAIKS  170

Query  173  DGNLTLMQKKVLVTQIGP----LKSSWRFTMDLPRDVTPEDIKKVL  214
               +T+  K++ V+   P    +K +  +  +LPR +T + +  + 
Sbjct  171  LNGITVRNKRLKVSYARPGGESIKDTNLYVTNLPRTITDDQLDTIF  216


>ref|XP_011176863.1| PREDICTED: sex-lethal homolog isoform X3 [Bactrocera cucurbitae]
Length=356

 Score = 42.0 bits (97),  Expect = 1.8, Method: Compositional matrix adjust.
 Identities = 29/106 (27%), Positives = 48/106 (45%), Gaps = 10/106 (9%)

Query  119  NILIEHLPPQMTKRELKALF------NELDIRVNSIVKIQNGRAFVALNGEHSLQRILAL  172
            N+++ +LP  MT REL ALF      N   I  +       G AFV    E   QR +  
Sbjct  119  NLIVNYLPQDMTDRELYALFRTIGPINTCRIMRDYKTGYSFGYAFVDFASERDSQRAIKS  178

Query  173  DGNLTLMQKKVLVTQIGP----LKSSWRFTMDLPRDVTPEDIKKVL  214
               +T+  K++ V+   P    +K +  +  +LPR +T + +  + 
Sbjct  179  LNGITVRNKRLKVSYARPGGESIKDTNLYVTNLPRTITDDQLDTIF  224


>ref|XP_013795280.1| PREDICTED: ELAV-like protein 2 isoform X1 [Apteryx australis 
mantelli]
Length=345

 Score = 41.6 bits (96),  Expect = 2.0, Method: Compositional matrix adjust.
 Identities = 37/143 (26%), Positives = 67/143 (47%), Gaps = 14/143 (10%)

Query  82   SRHPTSYTPWNKSNDKHIASAPLNTNNVSEEKARELCNILIEHLPPQMTKRELKALFNEL  141
            S  PT     N S   +  S+P+++ NV + K     N+++ +LP  MT+ ELK+LF  +
Sbjct  6    SNGPTCNNTANGSTAINNCSSPVDSGNVEDSKT----NLIVNYLPQNMTQEELKSLFGSI  61

Query  142  -DIRVNSIV--KIQN---GRAFVALNGEHSLQRILALDGNLTLMQKKVLVTQIGPLKSSW  195
             +I    +V  KI     G  FV        ++ +     L L  K + V+   P  +S 
Sbjct  62   GEIESCKLVRDKITGQSLGYGFVNYVDPKDAEKAINTLNGLRLQTKTIKVSYARPSSASI  121

Query  196  R----FTMDLPRDVTPEDIKKVL  214
            R    +   LP+ +T ++++++ 
Sbjct  122  RDANLYVSGLPKTMTQKELEQLF  144


>ref|XP_004391060.1| PREDICTED: uncharacterized protein C6orf10 homolog, partial [Trichechus 
manatus latirostris]
Length=399

 Score = 41.6 bits (96),  Expect = 2.2, Method: Compositional matrix adjust.
 Identities = 28/87 (32%), Positives = 43/87 (49%), Gaps = 1/87 (1%)

Query  238  VLQNDKGFLSRRSHRTSIRDSPNLSYGIRKYSTNVSVEEDDEFLRKGRRVRRPTEDIGVD  297
             + + +G L  RS + SI  + +   G  K  T V VEE D  + KG+  +    D G+ 
Sbjct  187  TIADSEGSLISRSKKESIGKAKSTELGKDKIETEVKVEESDAGILKGKEAQVKKSDTGIL  246

Query  298  PGPLSEDSPRSSTNHPSSEESPAQHSE  324
             GP S+   +S T+ P  +ES  Q S+
Sbjct  247  KGPDSQ-VKKSDTSIPKGQESQGQESD  272


>emb|CAG29242.1| sex-lethal protein [Bactrocera oleae]
Length=339

 Score = 41.6 bits (96),  Expect = 2.4, Method: Compositional matrix adjust.
 Identities = 35/137 (26%), Positives = 59/137 (43%), Gaps = 12/137 (9%)

Query  119  NILIEHLPPQMTKRELKALF------NELDIRVNSIVKIQNGRAFVALNGEHSLQRILAL  172
            N+++ +LP  MT REL ALF      N   I  +       G AFV    E   QR +  
Sbjct  106  NLIVNYLPQDMTDRELYALFRTIGPINTCRIMRDYKTGYSFGYAFVDFASETDSQRAIKS  165

Query  173  DGNLTLMQKKVLVTQIGP----LKSSWRFTMDLPRDVTPEDIKKVLQPLNVDILYN--KK  226
               +T+  K++ V+   P    +K +  +  +LPR +T + +  +     + +  N  + 
Sbjct  166  LNGITVRNKRLKVSYARPGGESIKDTNLYVTNLPRTITDDQLDTIFGKYGMIVQKNILRD  225

Query  227  LPVGEPNGDKRVLQNDK  243
               G+P G   V  N +
Sbjct  226  KLTGKPRGVAFVRFNKR  242


>ref|XP_013795281.1| PREDICTED: ELAV-like protein 2 isoform X2 [Apteryx australis 
mantelli]
Length=359

 Score = 41.6 bits (96),  Expect = 2.5, Method: Compositional matrix adjust.
 Identities = 37/143 (26%), Positives = 67/143 (47%), Gaps = 14/143 (10%)

Query  82   SRHPTSYTPWNKSNDKHIASAPLNTNNVSEEKARELCNILIEHLPPQMTKRELKALFNEL  141
            S  PT     N S   +  S+P+++ NV + K     N+++ +LP  MT+ ELK+LF  +
Sbjct  6    SNGPTCNNTANGSTAINNCSSPVDSGNVEDSKT----NLIVNYLPQNMTQEELKSLFGSI  61

Query  142  -DIRVNSIV--KIQN---GRAFVALNGEHSLQRILALDGNLTLMQKKVLVTQIGPLKSSW  195
             +I    +V  KI     G  FV        ++ +     L L  K + V+   P  +S 
Sbjct  62   GEIESCKLVRDKITGQSLGYGFVNYVDPKDAEKAINTLNGLRLQTKTIKVSYARPSSASI  121

Query  196  R----FTMDLPRDVTPEDIKKVL  214
            R    +   LP+ +T ++++++ 
Sbjct  122  RDANLYVSGLPKTMTQKELEQLF  144


>ref|XP_013795282.1| PREDICTED: ELAV-like protein 2 isoform X3 [Apteryx australis 
mantelli]
Length=358

 Score = 41.6 bits (96),  Expect = 2.5, Method: Compositional matrix adjust.
 Identities = 37/143 (26%), Positives = 67/143 (47%), Gaps = 14/143 (10%)

Query  82   SRHPTSYTPWNKSNDKHIASAPLNTNNVSEEKARELCNILIEHLPPQMTKRELKALFNEL  141
            S  PT     N S   +  S+P+++ NV + K     N+++ +LP  MT+ ELK+LF  +
Sbjct  6    SNGPTCNNTANGSTAINNCSSPVDSGNVEDSKT----NLIVNYLPQNMTQEELKSLFGSI  61

Query  142  -DIRVNSIV--KIQN---GRAFVALNGEHSLQRILALDGNLTLMQKKVLVTQIGPLKSSW  195
             +I    +V  KI     G  FV        ++ +     L L  K + V+   P  +S 
Sbjct  62   GEIESCKLVRDKITGQSLGYGFVNYVDPKDAEKAINTLNGLRLQTKTIKVSYARPSSASI  121

Query  196  R----FTMDLPRDVTPEDIKKVL  214
            R    +   LP+ +T ++++++ 
Sbjct  122  RDANLYVSGLPKTMTQKELEQLF  144


>ref|WP_048476863.1| peptidase M28 [Streptomyces roseus]
 gb|KMO97319.1| peptidase M28 [Streptomyces roseus]
Length=1158

 Score = 42.0 bits (97),  Expect = 2.7, Method: Compositional matrix adjust.
 Identities = 21/61 (34%), Positives = 31/61 (51%), Gaps = 2/61 (3%)

Query  47   IFVNRPPPFDKNDYLLPSMVNKT--EPIRNSRSSEASSRHPTSYTPWNKSNDKHIASAPL  104
            ++ N P P+D  DY +  M+N T   PIRN  +  A +  P  Y+      + H A+ PL
Sbjct  355  VYANEPAPYDTPDYTVGEMINLTGNGPIRNMYNPSAVNNDPNCYSSAIPGTEVHAAAGPL  414

Query  105  N  105
            N
Sbjct  415  N  415


>ref|XP_009685069.1| PREDICTED: ELAV-like protein 2 isoform X1 [Struthio camelus australis]
 ref|XP_009685072.1| PREDICTED: ELAV-like protein 2 isoform X1 [Struthio camelus australis]
Length=388

 Score = 41.6 bits (96),  Expect = 2.7, Method: Compositional matrix adjust.
 Identities = 37/143 (26%), Positives = 67/143 (47%), Gaps = 14/143 (10%)

Query  82   SRHPTSYTPWNKSNDKHIASAPLNTNNVSEEKARELCNILIEHLPPQMTKRELKALFNEL  141
            S  PT     N SN  +  S+P+++ N  + K     N+++ +LP  MT+ ELK+LF  +
Sbjct  35   SNGPTCNNTANGSNAINNCSSPVDSGNTEDSKT----NLIVNYLPQNMTQEELKSLFGSI  90

Query  142  -DIRVNSIV--KIQN---GRAFVALNGEHSLQRILALDGNLTLMQKKVLVTQIGPLKSSW  195
             +I    +V  KI     G  FV        ++ +     L L  K + V+   P  +S 
Sbjct  91   GEIESCKLVRDKITGQSLGYGFVNYVDPKDAEKAINTLNGLRLQTKTIKVSYARPSSASI  150

Query  196  R----FTMDLPRDVTPEDIKKVL  214
            R    +   LP+ +T ++++++ 
Sbjct  151  RDANLYVSGLPKTMTQKELEQLF  173


>ref|XP_009685070.1| PREDICTED: ELAV-like protein 2 isoform X2 [Struthio camelus australis]
Length=387

 Score = 41.2 bits (95),  Expect = 2.8, Method: Compositional matrix adjust.
 Identities = 37/143 (26%), Positives = 67/143 (47%), Gaps = 14/143 (10%)

Query  82   SRHPTSYTPWNKSNDKHIASAPLNTNNVSEEKARELCNILIEHLPPQMTKRELKALFNEL  141
            S  PT     N SN  +  S+P+++ N  + K     N+++ +LP  MT+ ELK+LF  +
Sbjct  34   SNGPTCNNTANGSNAINNCSSPVDSGNTEDSKT----NLIVNYLPQNMTQEELKSLFGSI  89

Query  142  -DIRVNSIV--KIQN---GRAFVALNGEHSLQRILALDGNLTLMQKKVLVTQIGPLKSSW  195
             +I    +V  KI     G  FV        ++ +     L L  K + V+   P  +S 
Sbjct  90   GEIESCKLVRDKITGQSLGYGFVNYVDPKDAEKAINTLNGLRLQTKTIKVSYARPSSASI  149

Query  196  R----FTMDLPRDVTPEDIKKVL  214
            R    +   LP+ +T ++++++ 
Sbjct  150  RDANLYVSGLPKTMTQKELEQLF  172


>ref|XP_011211864.1| PREDICTED: sex-lethal homolog isoform X1 [Bactrocera dorsalis]
Length=354

 Score = 41.2 bits (95),  Expect = 2.9, Method: Compositional matrix adjust.
 Identities = 32/107 (30%), Positives = 52/107 (49%), Gaps = 12/107 (11%)

Query  119  NILIEHLPPQMTKRELKALF------NELDIRVNSIVKIQNGRAFVALNGEHSLQR-ILA  171
            N+++ +LP  MT REL ALF      N   I  +       G AFV    E   QR I +
Sbjct  122  NLIVNYLPQDMTDRELYALFRTIGPINTCRIMRDYKTGYSFGYAFVDYASETDSQRAIRS  181

Query  172  LDGNLTLMQKKVLVTQIGP----LKSSWRFTMDLPRDVTPEDIKKVL  214
            L+G +T+  K++ V+   P    +K +  +  +LPR +T + +  + 
Sbjct  182  LNG-ITVRNKRLKVSYARPGGESIKDTNLYVTNLPRTITDDQLDTIF  227


>ref|XP_011176861.1| PREDICTED: sex-lethal homolog isoform X1 [Bactrocera cucurbitae]
Length=370

 Score = 41.2 bits (95),  Expect = 2.9, Method: Compositional matrix adjust.
 Identities = 29/106 (27%), Positives = 48/106 (45%), Gaps = 10/106 (9%)

Query  119  NILIEHLPPQMTKRELKALF------NELDIRVNSIVKIQNGRAFVALNGEHSLQRILAL  172
            N+++ +LP  MT REL ALF      N   I  +       G AFV    E   QR +  
Sbjct  119  NLIVNYLPQDMTDRELYALFRTIGPINTCRIMRDYKTGYSFGYAFVDFASERDSQRAIKS  178

Query  173  DGNLTLMQKKVLVTQIGP----LKSSWRFTMDLPRDVTPEDIKKVL  214
               +T+  K++ V+   P    +K +  +  +LPR +T + +  + 
Sbjct  179  LNGITVRNKRLKVSYARPGGESIKDTNLYVTNLPRTITDDQLDTIF  224


>ref|XP_009685073.1| PREDICTED: ELAV-like protein 2 isoform X4 [Struthio camelus australis]
Length=387

 Score = 41.2 bits (95),  Expect = 2.9, Method: Compositional matrix adjust.
 Identities = 37/143 (26%), Positives = 67/143 (47%), Gaps = 14/143 (10%)

Query  82   SRHPTSYTPWNKSNDKHIASAPLNTNNVSEEKARELCNILIEHLPPQMTKRELKALFNEL  141
            S  PT     N SN  +  S+P+++ N  + K     N+++ +LP  MT+ ELK+LF  +
Sbjct  35   SNGPTCNNTANGSNAINNCSSPVDSGNTEDSKT----NLIVNYLPQNMTQEELKSLFGSI  90

Query  142  -DIRVNSIV--KIQN---GRAFVALNGEHSLQRILALDGNLTLMQKKVLVTQIGPLKSSW  195
             +I    +V  KI     G  FV        ++ +     L L  K + V+   P  +S 
Sbjct  91   GEIESCKLVRDKITGQSLGYGFVNYVDPKDAEKAINTLNGLRLQTKTIKVSYARPSSASI  150

Query  196  R----FTMDLPRDVTPEDIKKVL  214
            R    +   LP+ +T ++++++ 
Sbjct  151  RDANLYVSGLPKTMTQKELEQLF  173


>ref|WP_026220098.1| peptidase M28 [Streptomyces vitaminophilus]
Length=1160

 Score = 41.6 bits (96),  Expect = 3.2, Method: Compositional matrix adjust.
 Identities = 22/61 (36%), Positives = 33/61 (54%), Gaps = 3/61 (5%)

Query  48   FVNRPPPFDK-NDYLLPSMVN--KTEPIRNSRSSEASSRHPTSYTPWNKSNDKHIASAPL  104
            + N P P+D   DYL+  M++   T PIRN  +    S HP  Y+    + ++H A+ PL
Sbjct  361  YANEPAPYDTPGDYLVGEMIDLVGTGPIRNMYNPSLVSGHPNCYSSSIPNTEEHAAAGPL  420

Query  105  N  105
            N
Sbjct  421  N  421


>ref|XP_007560453.1| PREDICTED: ribonucleoprotein PTB-binding 1 isoform X1 [Poecilia 
formosa]
Length=747

 Score = 41.6 bits (96),  Expect = 3.3, Method: Compositional matrix adjust.
 Identities = 46/186 (25%), Positives = 83/186 (45%), Gaps = 12/186 (6%)

Query  39   DDSARASPIFVNRPPPFDKNDYLLPSMVNKTEPIRNSRSSEASSRHPTSYTP--WNKSND  96
            D+ A +   FV+RP  F  N  L     N     R      AS     +  P    + ++
Sbjct  14   DEGADSGATFVSRP--FSANHDLATDKENFMAGDRQRYPELASVEEDATSGPECQRRVDE  71

Query  97   KHIASAPLNTNNVSEEKARELCN---ILIEHLPPQMTKRELKALFNELDIRVNSIVKIQN  153
              I  +P +  +  E   RE  N   I+I++LPP +T +E+  L    D++   + K + 
Sbjct  72   DLIPLSPEDIESRLERTRREFYNRRKIIIKNLPPDVTNQEVHDLLGNYDLKYCFVDKYK-  130

Query  154  GRAFVA-LNGEHSLQRILALDGNLTLMQKKVLVTQIGPLKSSWRFTMDLPRDVTPEDIKK  212
            G AFV  LNGE +   I     +  +++++ +  Q+ P   +     +LPR  T +  ++
Sbjct  131  GTAFVTLLNGEQAQCAIKEF--HQIILREREISVQLQP-TDALLCIANLPRAFTQQQFEE  187

Query  213  VLQPLN  218
            +++P  
Sbjct  188  LVRPFG  193


>ref|XP_007560454.1| PREDICTED: ribonucleoprotein PTB-binding 1 isoform X2 [Poecilia 
formosa]
Length=735

 Score = 41.6 bits (96),  Expect = 3.3, Method: Compositional matrix adjust.
 Identities = 46/186 (25%), Positives = 83/186 (45%), Gaps = 12/186 (6%)

Query  39   DDSARASPIFVNRPPPFDKNDYLLPSMVNKTEPIRNSRSSEASSRHPTSYTP--WNKSND  96
            D+ A +   FV+RP  F  N  L     N     R      AS     +  P    + ++
Sbjct  14   DEGADSGATFVSRP--FSANHDLATDKENFMAGDRQRYPELASVEEDATSGPECQRRVDE  71

Query  97   KHIASAPLNTNNVSEEKARELCN---ILIEHLPPQMTKRELKALFNELDIRVNSIVKIQN  153
              I  +P +  +  E   RE  N   I+I++LPP +T +E+  L    D++   + K + 
Sbjct  72   DLIPLSPEDIESRLERTRREFYNRRKIIIKNLPPDVTNQEVHDLLGNYDLKYCFVDKYK-  130

Query  154  GRAFVA-LNGEHSLQRILALDGNLTLMQKKVLVTQIGPLKSSWRFTMDLPRDVTPEDIKK  212
            G AFV  LNGE +   I     +  +++++ +  Q+ P   +     +LPR  T +  ++
Sbjct  131  GTAFVTLLNGEQAQCAIKEF--HQIILREREISVQLQP-TDALLCIANLPRAFTQQQFEE  187

Query  213  VLQPLN  218
            +++P  
Sbjct  188  LVRPFG  193


>ref|XP_011176862.1| PREDICTED: sex-lethal homolog isoform X2 [Bactrocera cucurbitae]
Length=362

 Score = 41.2 bits (95),  Expect = 3.5, Method: Compositional matrix adjust.
 Identities = 29/106 (27%), Positives = 48/106 (45%), Gaps = 10/106 (9%)

Query  119  NILIEHLPPQMTKRELKALF------NELDIRVNSIVKIQNGRAFVALNGEHSLQRILAL  172
            N+++ +LP  MT REL ALF      N   I  +       G AFV    E   QR +  
Sbjct  111  NLIVNYLPQDMTDRELYALFRTIGPINTCRIMRDYKTGYSFGYAFVDFASERDSQRAIKS  170

Query  173  DGNLTLMQKKVLVTQIGP----LKSSWRFTMDLPRDVTPEDIKKVL  214
               +T+  K++ V+   P    +K +  +  +LPR +T + +  + 
Sbjct  171  LNGITVRNKRLKVSYARPGGESIKDTNLYVTNLPRTITDDQLDTIF  216


>gb|AIK25412.1| sex-lethal-like protein, partial [Bactrocera jarvisi]
Length=307

 Score = 40.8 bits (94),  Expect = 3.5, Method: Compositional matrix adjust.
 Identities = 32/107 (30%), Positives = 52/107 (49%), Gaps = 12/107 (11%)

Query  119  NILIEHLPPQMTKRELKALF------NELDIRVNSIVKIQNGRAFVALNGEHSLQR-ILA  171
            N+++ +LP  MT REL ALF      N   I  +       G AFV    E   QR I +
Sbjct  76   NLIVNYLPQDMTDRELYALFRTIGPINTCRIMRDYKTGYSFGYAFVDYASETDSQRAIRS  135

Query  172  LDGNLTLMQKKVLVTQIGP----LKSSWRFTMDLPRDVTPEDIKKVL  214
            L+G +T+  K++ V+   P    +K +  +  +LPR +T + +  + 
Sbjct  136  LNG-ITVRNKRLKVSYARPGGESIKDTNLYVTNLPRTITDDQLDTIF  181


>ref|XP_011211868.1| PREDICTED: sex-lethal homolog isoform X3 [Bactrocera dorsalis]
Length=353

 Score = 40.8 bits (94),  Expect = 3.6, Method: Compositional matrix adjust.
 Identities = 38/138 (28%), Positives = 63/138 (46%), Gaps = 14/138 (10%)

Query  119  NILIEHLPPQMTKRELKALF------NELDIRVNSIVKIQNGRAFVALNGEHSLQR-ILA  171
            N+++ +LP  MT REL ALF      N   I  +       G AFV    E   QR I +
Sbjct  122  NLIVNYLPQDMTDRELYALFRTIGPINTCRIMRDYKTGYSFGYAFVDYASETDSQRAIRS  181

Query  172  LDGNLTLMQKKVLVTQIGP----LKSSWRFTMDLPRDVTPEDIKKVLQPLNVDILYN--K  225
            L+G +T+  K++ V+   P    +K +  +  +LPR +T + +  +     + +  N  +
Sbjct  182  LNG-ITVRNKRLKVSYARPGGESIKDTNLYVTNLPRTITDDQLDTIFGKYGMIVQKNILR  240

Query  226  KLPVGEPNGDKRVLQNDK  243
                G+P G   V  N +
Sbjct  241  DKLTGKPRGVAFVRFNKR  258


>ref|XP_011211869.1| PREDICTED: sex-lethal homolog isoform X4 [Bactrocera dorsalis]
Length=346

 Score = 40.8 bits (94),  Expect = 3.6, Method: Compositional matrix adjust.
 Identities = 32/107 (30%), Positives = 52/107 (49%), Gaps = 12/107 (11%)

Query  119  NILIEHLPPQMTKRELKALF------NELDIRVNSIVKIQNGRAFVALNGEHSLQR-ILA  171
            N+++ +LP  MT REL ALF      N   I  +       G AFV    E   QR I +
Sbjct  114  NLIVNYLPQDMTDRELYALFRTIGPINTCRIMRDYKTGYSFGYAFVDYASETDSQRAIRS  173

Query  172  LDGNLTLMQKKVLVTQIGP----LKSSWRFTMDLPRDVTPEDIKKVL  214
            L+G +T+  K++ V+   P    +K +  +  +LPR +T + +  + 
Sbjct  174  LNG-ITVRNKRLKVSYARPGGESIKDTNLYVTNLPRTITDDQLDTIF  219


>emb|CDS26875.1| ELAV protein 2 [Hymenolepis microstoma]
Length=577

 Score = 41.2 bits (95),  Expect = 4.1, Method: Compositional matrix adjust.
 Identities = 28/121 (23%), Positives = 59/121 (49%), Gaps = 12/121 (10%)

Query  105  NTNNVSEEKARELCNILIEHLPPQMTKRELKALFNELDIRVNSIVKIQN-------GRAF  157
            ++ N SE       N+++ +LP  M + E++ALFN +  +V+S   I++       G  F
Sbjct  100  SSQNSSETTTENKTNLIVNYLPQSMNQEEIRALFNTIG-KVSSCKLIRDKSTGQSLGYGF  158

Query  158  VALNGEHSLQRILALDGNLTLMQKKVLVTQIGP----LKSSWRFTMDLPRDVTPEDIKKV  213
            V        ++ +     + L  KK+ V+   P    +K +  +   LP+++T ++++K+
Sbjct  159  VNYANASDAEKAIKHFNGMRLKNKKIKVSLARPSSESIKGANLYICGLPKNITQDELEKI  218

Query  214  L  214
             
Sbjct  219  F  219


>ref|XP_011211865.1| PREDICTED: sex-lethal homolog isoform X2 [Bactrocera dorsalis]
Length=354

 Score = 40.8 bits (94),  Expect = 4.4, Method: Compositional matrix adjust.
 Identities = 35/137 (26%), Positives = 59/137 (43%), Gaps = 12/137 (9%)

Query  119  NILIEHLPPQMTKRELKALF------NELDIRVNSIVKIQNGRAFVALNGEHSLQRILAL  172
            N+++ +LP  MT REL ALF      N   I  +       G AFV    E   QR +  
Sbjct  122  NLIVNYLPQDMTDRELYALFRTIGPINTCRIMRDYKTGYSFGYAFVDYASETDSQRAIRS  181

Query  173  DGNLTLMQKKVLVTQIGP----LKSSWRFTMDLPRDVTPEDIKKVLQPLNVDILYN--KK  226
               +T+  K++ V+   P    +K +  +  +LPR +T + +  +     + +  N  + 
Sbjct  182  LNGITVRNKRLKVSYARPGGESIKDTNLYVTNLPRTITDDQLDTIFGKYGMIVQKNILRD  241

Query  227  LPVGEPNGDKRVLQNDK  243
               G+P G   V  N +
Sbjct  242  KLTGKPRGVAFVRFNKR  258


>ref|XP_010724453.1| PREDICTED: ELAV-like protein 2 isoform X2 [Meleagris gallopavo]
Length=278

 Score = 40.4 bits (93),  Expect = 4.6, Method: Compositional matrix adjust.
 Identities = 43/175 (25%), Positives = 76/175 (43%), Gaps = 22/175 (13%)

Query  82   SRHPTSYTPWNKSNDKHIASAPLNTNNVSEEKARELCNILIEHLPPQMTKRELKALFNEL  141
            S  PT     + SN  +  S+P+++ N  + K     N+++ +LP  MT+ ELK+LF  +
Sbjct  34   SNGPTCNNTAHGSNTINNCSSPIDSGNAEDSKT----NLIVNYLPQSMTQEELKSLFGSI  89

Query  142  -DIRVNSIV--KIQN---GRAFVALNGEHSLQRILALDGNLTLMQKKVLVTQIGPLKSSW  195
             +I    +V  KI     G  FV        ++ +     L L  K + V+   P  +S 
Sbjct  90   GEIESCKLVRDKITGQSLGYGFVNYVDPKDAEKAINTLNGLRLQTKTIKVSYARPSSASI  149

Query  196  R----FTMDLPRDVTPEDIKKVLQPLN--------VDILYNKKLPVGEPNGDKRV  238
            R    +   LP+ +T ++++++             VD +      VG    DKR+
Sbjct  150  RDANLYVSGLPKTMTQKELEQLFSQYGRIITSRILVDQVTGMSRGVGFIRFDKRI  204


>ref|XP_010724452.1| PREDICTED: ELAV-like protein 2 isoform X1 [Meleagris gallopavo]
Length=279

 Score = 40.4 bits (93),  Expect = 4.6, Method: Compositional matrix adjust.
 Identities = 43/175 (25%), Positives = 76/175 (43%), Gaps = 22/175 (13%)

Query  82   SRHPTSYTPWNKSNDKHIASAPLNTNNVSEEKARELCNILIEHLPPQMTKRELKALFNEL  141
            S  PT     + SN  +  S+P+++ N  + K     N+++ +LP  MT+ ELK+LF  +
Sbjct  35   SNGPTCNNTAHGSNTINNCSSPIDSGNAEDSKT----NLIVNYLPQSMTQEELKSLFGSI  90

Query  142  -DIRVNSIV--KIQN---GRAFVALNGEHSLQRILALDGNLTLMQKKVLVTQIGPLKSSW  195
             +I    +V  KI     G  FV        ++ +     L L  K + V+   P  +S 
Sbjct  91   GEIESCKLVRDKITGQSLGYGFVNYVDPKDAEKAINTLNGLRLQTKTIKVSYARPSSASI  150

Query  196  R----FTMDLPRDVTPEDIKKVLQPLN--------VDILYNKKLPVGEPNGDKRV  238
            R    +   LP+ +T ++++++             VD +      VG    DKR+
Sbjct  151  RDANLYVSGLPKTMTQKELEQLFSQYGRIITSRILVDQVTGMSRGVGFIRFDKRI  205


>ref|XP_011211870.1| PREDICTED: sex-lethal homolog isoform X5 [Bactrocera dorsalis]
Length=345

 Score = 40.4 bits (93),  Expect = 4.7, Method: Compositional matrix adjust.
 Identities = 32/107 (30%), Positives = 52/107 (49%), Gaps = 12/107 (11%)

Query  119  NILIEHLPPQMTKRELKALF------NELDIRVNSIVKIQNGRAFVALNGEHSLQR-ILA  171
            N+++ +LP  MT REL ALF      N   I  +       G AFV    E   QR I +
Sbjct  114  NLIVNYLPQDMTDRELYALFRTIGPINTCRIMRDYKTGYSFGYAFVDYASETDSQRAIRS  173

Query  172  LDGNLTLMQKKVLVTQIGP----LKSSWRFTMDLPRDVTPEDIKKVL  214
            L+G +T+  K++ V+   P    +K +  +  +LPR +T + +  + 
Sbjct  174  LNG-ITVRNKRLKVSYARPGGESIKDTNLYVTNLPRTITDDQLDTIF  219


>ref|WP_023109419.1| hypothetical protein [Pseudomonas aeruginosa]
 gb|ERY58739.1| hypothetical protein Q056_03518 [Pseudomonas aeruginosa BL02]
Length=127

 Score = 38.5 bits (88),  Expect = 5.1, Method: Composition-based stats.
 Identities = 28/70 (40%), Positives = 36/70 (51%), Gaps = 4/70 (6%)

Query  71   PIRNSRSSEASSRHPTSYTPWNKSNDKHIASAPLNTNNVSEEKARELCNILIEHLPPQMT  130
            P R  RSS+ ++ H  S   WN   D+    A LN   VSE   R LC  L+ H    + 
Sbjct  55   PDRLPRSSDIAAIHQHS-IGWNACLDE---LARLNGLTVSEGLLRRLCEPLLSHADYVVV  110

Query  131  KRELKALFNE  140
            + EL+ALFNE
Sbjct  111  RDELRALFNE  120


>ref|XP_005499263.1| PREDICTED: ELAV-like protein 2 isoform X3 [Columba livia]
Length=345

 Score = 40.4 bits (93),  Expect = 5.2, Method: Compositional matrix adjust.
 Identities = 36/143 (25%), Positives = 67/143 (47%), Gaps = 14/143 (10%)

Query  82   SRHPTSYTPWNKSNDKHIASAPLNTNNVSEEKARELCNILIEHLPPQMTKRELKALFNEL  141
            S  PT     + SN  +  S+P+++ N  + K     N+++ +LP  MT+ ELK+LF  +
Sbjct  6    SNGPTCNNTAHSSNTINNCSSPVDSGNTEDSKT----NLIVNYLPQNMTQEELKSLFGSI  61

Query  142  -DIRVNSIV--KIQN---GRAFVALNGEHSLQRILALDGNLTLMQKKVLVTQIGPLKSSW  195
             +I    +V  KI     G  FV        ++ +     L L  K + V+   P  +S 
Sbjct  62   GEIESCKLVRDKITGQSLGYGFVNYVDPKDAEKAINTLNGLRLQTKTIKVSYARPSSASI  121

Query  196  R----FTMDLPRDVTPEDIKKVL  214
            R    +   LP+ +T ++++++ 
Sbjct  122  RDANLYVSGLPKTMTQKELEQLF  144


>ref|XP_013225007.1| PREDICTED: ELAV-like protein 2 isoform X1 [Columba livia]
Length=346

 Score = 40.4 bits (93),  Expect = 5.3, Method: Compositional matrix adjust.
 Identities = 36/143 (25%), Positives = 67/143 (47%), Gaps = 14/143 (10%)

Query  82   SRHPTSYTPWNKSNDKHIASAPLNTNNVSEEKARELCNILIEHLPPQMTKRELKALFNEL  141
            S  PT     + SN  +  S+P+++ N  + K     N+++ +LP  MT+ ELK+LF  +
Sbjct  6    SNGPTCNNTAHSSNTINNCSSPVDSGNTEDSKT----NLIVNYLPQNMTQEELKSLFGSI  61

Query  142  -DIRVNSIV--KIQN---GRAFVALNGEHSLQRILALDGNLTLMQKKVLVTQIGPLKSSW  195
             +I    +V  KI     G  FV        ++ +     L L  K + V+   P  +S 
Sbjct  62   GEIESCKLVRDKITGQSLGYGFVNYVDPKDAEKAINTLNGLRLQTKTIKVSYARPSSASI  121

Query  196  R----FTMDLPRDVTPEDIKKVL  214
            R    +   LP+ +T ++++++ 
Sbjct  122  RDANLYVSGLPKTMTQKELEQLF  144


>ref|XP_004949599.1| PREDICTED: ELAV-like protein 2 isoform X8 [Gallus gallus]
Length=437

 Score = 40.4 bits (93),  Expect = 5.5, Method: Compositional matrix adjust.
 Identities = 43/175 (25%), Positives = 76/175 (43%), Gaps = 22/175 (13%)

Query  82   SRHPTSYTPWNKSNDKHIASAPLNTNNVSEEKARELCNILIEHLPPQMTKRELKALFNEL  141
            S  PT     + SN  +  S+P+++ N  + K     N+++ +LP  MT+ ELK+LF  +
Sbjct  84   SNGPTCNNTAHGSNAINNCSSPIDSGNTEDSKT----NLIVNYLPQSMTQEELKSLFGSI  139

Query  142  -DIRVNSIV--KIQN---GRAFVALNGEHSLQRILALDGNLTLMQKKVLVTQIGPLKSSW  195
             +I    +V  KI     G  FV        ++ +     L L  K + V+   P  +S 
Sbjct  140  GEIESCKLVRDKITGQSLGYGFVNYVDPKDAEKAINTLNGLRLQTKTIKVSYARPSSASI  199

Query  196  R----FTMDLPRDVTPEDIKKVLQPLN--------VDILYNKKLPVGEPNGDKRV  238
            R    +   LP+ +T ++++++             VD +      VG    DKR+
Sbjct  200  RDANLYVSGLPKTMTQKELEQLFSQYGRIITSRILVDQVTGMSRGVGFIRFDKRI  254


>ref|XP_009965948.1| PREDICTED: ELAV-like protein 2 isoform X3 [Tyto alba]
Length=371

 Score = 40.4 bits (93),  Expect = 5.5, Method: Compositional matrix adjust.
 Identities = 39/144 (27%), Positives = 73/144 (51%), Gaps = 16/144 (11%)

Query  82   SRHPTSYTPWNKSNDKHIASAPLNTNNVSEEKARELCNILIEHLPPQMTKRELKALFNEL  141
            S  PT     + S   +  S+P+N+ N  + K     N+++ +LP  MT+ ELK+LF  +
Sbjct  6    SNGPTCNNTAHSSTTVNNCSSPVNSGNTEDSKT----NLIVNYLPQNMTQEELKSLFGSI  61

Query  142  -DIRVNSIV--KIQN---GRAFV-ALNGEHSLQRILALDGNLTLMQKKVLVTQIGPLKSS  194
             +I    +V  KI     G  FV  ++ +++ + I  L+G L L  K + V+   P  +S
Sbjct  62   GEIESCKLVRDKITGQSLGYGFVNYVDPKNAEKAINTLNG-LRLQTKTIKVSYARPSSAS  120

Query  195  WR----FTMDLPRDVTPEDIKKVL  214
             R    +   LP+ +T ++++++ 
Sbjct  121  IRDANLYVSGLPKTMTQKELEQLF  144


>ref|XP_004949598.1| PREDICTED: ELAV-like protein 2 isoform X7 [Gallus gallus]
Length=437

 Score = 40.4 bits (93),  Expect = 5.8, Method: Compositional matrix adjust.
 Identities = 43/175 (25%), Positives = 76/175 (43%), Gaps = 22/175 (13%)

Query  82   SRHPTSYTPWNKSNDKHIASAPLNTNNVSEEKARELCNILIEHLPPQMTKRELKALFNEL  141
            S  PT     + SN  +  S+P+++ N  + K     N+++ +LP  MT+ ELK+LF  +
Sbjct  97   SNGPTCNNTAHGSNAINNCSSPIDSGNTEDSKT----NLIVNYLPQSMTQEELKSLFGSI  152

Query  142  -DIRVNSIV--KIQN---GRAFVALNGEHSLQRILALDGNLTLMQKKVLVTQIGPLKSSW  195
             +I    +V  KI     G  FV        ++ +     L L  K + V+   P  +S 
Sbjct  153  GEIESCKLVRDKITGQSLGYGFVNYVDPKDAEKAINTLNGLRLQTKTIKVSYARPSSASI  212

Query  196  R----FTMDLPRDVTPEDIKKVLQPLN--------VDILYNKKLPVGEPNGDKRV  238
            R    +   LP+ +T ++++++             VD +      VG    DKR+
Sbjct  213  RDANLYVSGLPKTMTQKELEQLFSQYGRIITSRILVDQVTGMSRGVGFIRFDKRI  267


>ref|XP_004949606.1| PREDICTED: ELAV-like protein 2 isoform X15 [Gallus gallus]
 ref|XP_004949607.1| PREDICTED: ELAV-like protein 2 isoform X16 [Gallus gallus]
Length=359

 Score = 40.4 bits (93),  Expect = 5.9, Method: Compositional matrix adjust.
 Identities = 43/175 (25%), Positives = 76/175 (43%), Gaps = 22/175 (13%)

Query  82   SRHPTSYTPWNKSNDKHIASAPLNTNNVSEEKARELCNILIEHLPPQMTKRELKALFNEL  141
            S  PT     + SN  +  S+P+++ N  + K     N+++ +LP  MT+ ELK+LF  +
Sbjct  6    SNGPTCNNTAHGSNAINNCSSPIDSGNTEDSKT----NLIVNYLPQSMTQEELKSLFGSI  61

Query  142  -DIRVNSIV--KIQN---GRAFVALNGEHSLQRILALDGNLTLMQKKVLVTQIGPLKSSW  195
             +I    +V  KI     G  FV        ++ +     L L  K + V+   P  +S 
Sbjct  62   GEIESCKLVRDKITGQSLGYGFVNYVDPKDAEKAINTLNGLRLQTKTIKVSYARPSSASI  121

Query  196  R----FTMDLPRDVTPEDIKKVLQPLN--------VDILYNKKLPVGEPNGDKRV  238
            R    +   LP+ +T ++++++             VD +      VG    DKR+
Sbjct  122  RDANLYVSGLPKTMTQKELEQLFSQYGRIITSRILVDQVTGMSRGVGFIRFDKRI  176


>ref|XP_013225008.1| PREDICTED: ELAV-like protein 2 isoform X2 [Columba livia]
Length=359

 Score = 40.4 bits (93),  Expect = 6.0, Method: Compositional matrix adjust.
 Identities = 36/143 (25%), Positives = 67/143 (47%), Gaps = 14/143 (10%)

Query  82   SRHPTSYTPWNKSNDKHIASAPLNTNNVSEEKARELCNILIEHLPPQMTKRELKALFNEL  141
            S  PT     + SN  +  S+P+++ N  + K     N+++ +LP  MT+ ELK+LF  +
Sbjct  6    SNGPTCNNTAHSSNTINNCSSPVDSGNTEDSKT----NLIVNYLPQNMTQEELKSLFGSI  61

Query  142  -DIRVNSIV--KIQN---GRAFVALNGEHSLQRILALDGNLTLMQKKVLVTQIGPLKSSW  195
             +I    +V  KI     G  FV        ++ +     L L  K + V+   P  +S 
Sbjct  62   GEIESCKLVRDKITGQSLGYGFVNYVDPKDAEKAINTLNGLRLQTKTIKVSYARPSSASI  121

Query  196  R----FTMDLPRDVTPEDIKKVL  214
            R    +   LP+ +T ++++++ 
Sbjct  122  RDANLYVSGLPKTMTQKELEQLF  144


>ref|XP_004949600.1| PREDICTED: ELAV-like protein 2 isoform X9 [Gallus gallus]
Length=436

 Score = 40.4 bits (93),  Expect = 6.2, Method: Compositional matrix adjust.
 Identities = 43/175 (25%), Positives = 76/175 (43%), Gaps = 22/175 (13%)

Query  82   SRHPTSYTPWNKSNDKHIASAPLNTNNVSEEKARELCNILIEHLPPQMTKRELKALFNEL  141
            S  PT     + SN  +  S+P+++ N  + K     N+++ +LP  MT+ ELK+LF  +
Sbjct  97   SNGPTCNNTAHGSNAINNCSSPIDSGNTEDSKT----NLIVNYLPQSMTQEELKSLFGSI  152

Query  142  -DIRVNSIV--KIQN---GRAFVALNGEHSLQRILALDGNLTLMQKKVLVTQIGPLKSSW  195
             +I    +V  KI     G  FV        ++ +     L L  K + V+   P  +S 
Sbjct  153  GEIESCKLVRDKITGQSLGYGFVNYVDPKDAEKAINTLNGLRLQTKTIKVSYARPSSASI  212

Query  196  R----FTMDLPRDVTPEDIKKVLQPLN--------VDILYNKKLPVGEPNGDKRV  238
            R    +   LP+ +T ++++++             VD +      VG    DKR+
Sbjct  213  RDANLYVSGLPKTMTQKELEQLFSQYGRIITSRILVDQVTGMSRGVGFIRFDKRI  267


>ref|XP_013225013.1| PREDICTED: ELAV-like protein 2 isoform X4 [Columba livia]
Length=364

 Score = 40.4 bits (93),  Expect = 6.2, Method: Compositional matrix adjust.
 Identities = 36/143 (25%), Positives = 67/143 (47%), Gaps = 14/143 (10%)

Query  82   SRHPTSYTPWNKSNDKHIASAPLNTNNVSEEKARELCNILIEHLPPQMTKRELKALFNEL  141
            S  PT     + SN  +  S+P+++ N  + K     N+++ +LP  MT+ ELK+LF  +
Sbjct  6    SNGPTCNNTAHSSNTINNCSSPVDSGNTEDSKT----NLIVNYLPQNMTQEELKSLFGSI  61

Query  142  -DIRVNSIV--KIQN---GRAFVALNGEHSLQRILALDGNLTLMQKKVLVTQIGPLKSSW  195
             +I    +V  KI     G  FV        ++ +     L L  K + V+   P  +S 
Sbjct  62   GEIESCKLVRDKITGQSLGYGFVNYVDPKDAEKAINTLNGLRLQTKTIKVSYARPSSASI  121

Query  196  R----FTMDLPRDVTPEDIKKVL  214
            R    +   LP+ +T ++++++ 
Sbjct  122  RDANLYVSGLPKTMTQKELEQLF  144


>ref|XP_013225015.1| PREDICTED: ELAV-like protein 2 isoform X5 [Columba livia]
Length=358

 Score = 40.0 bits (92),  Expect = 6.5, Method: Compositional matrix adjust.
 Identities = 36/143 (25%), Positives = 67/143 (47%), Gaps = 14/143 (10%)

Query  82   SRHPTSYTPWNKSNDKHIASAPLNTNNVSEEKARELCNILIEHLPPQMTKRELKALFNEL  141
            S  PT     + SN  +  S+P+++ N  + K     N+++ +LP  MT+ ELK+LF  +
Sbjct  6    SNGPTCNNTAHSSNTINNCSSPVDSGNTEDSKT----NLIVNYLPQNMTQEELKSLFGSI  61

Query  142  -DIRVNSIV--KIQN---GRAFVALNGEHSLQRILALDGNLTLMQKKVLVTQIGPLKSSW  195
             +I    +V  KI     G  FV        ++ +     L L  K + V+   P  +S 
Sbjct  62   GEIESCKLVRDKITGQSLGYGFVNYVDPKDAEKAINTLNGLRLQTKTIKVSYARPSSASI  121

Query  196  R----FTMDLPRDVTPEDIKKVL  214
            R    +   LP+ +T ++++++ 
Sbjct  122  RDANLYVSGLPKTMTQKELEQLF  144


>ref|XP_009965947.1| PREDICTED: ELAV-like protein 2 isoform X2 [Tyto alba]
Length=374

 Score = 40.0 bits (92),  Expect = 6.5, Method: Compositional matrix adjust.
 Identities = 46/176 (26%), Positives = 82/176 (47%), Gaps = 24/176 (14%)

Query  82   SRHPTSYTPWNKSNDKHIASAPLNTNNVSEEKARELCNILIEHLPPQMTKRELKALFNEL  141
            S  PT     + S   +  S+P+N+ N  + K     N+++ +LP  MT+ ELK+LF  +
Sbjct  35   SNGPTCNNTAHSSTTVNNCSSPVNSGNTEDSKT----NLIVNYLPQNMTQEELKSLFGSI  90

Query  142  -DIRVNSIV--KIQN---GRAFV-ALNGEHSLQRILALDGNLTLMQKKVLVTQIGPLKSS  194
             +I    +V  KI     G  FV  ++ +++ + I  L+G L L  K + V+   P  +S
Sbjct  91   GEIESCKLVRDKITGQSLGYGFVNYVDPKNAEKAINTLNG-LRLQTKTIKVSYARPSSAS  149

Query  195  WR----FTMDLPRDVTPEDIKKVLQPLN--------VDILYNKKLPVGEPNGDKRV  238
             R    +   LP+ +T ++++++             VD +      VG    DKR+
Sbjct  150  IRDANLYVSGLPKTMTQKELEQLFSQYGRIITSRILVDQVTGVSRGVGFIRFDKRI  205


>ref|XP_004949595.1| PREDICTED: ELAV-like protein 2 isoform X4 [Gallus gallus]
Length=449

 Score = 40.4 bits (93),  Expect = 6.7, Method: Compositional matrix adjust.
 Identities = 43/175 (25%), Positives = 76/175 (43%), Gaps = 22/175 (13%)

Query  82   SRHPTSYTPWNKSNDKHIASAPLNTNNVSEEKARELCNILIEHLPPQMTKRELKALFNEL  141
            S  PT     + SN  +  S+P+++ N  + K     N+++ +LP  MT+ ELK+LF  +
Sbjct  96   SNGPTCNNTAHGSNAINNCSSPIDSGNTEDSKT----NLIVNYLPQSMTQEELKSLFGSI  151

Query  142  -DIRVNSIV--KIQN---GRAFVALNGEHSLQRILALDGNLTLMQKKVLVTQIGPLKSSW  195
             +I    +V  KI     G  FV        ++ +     L L  K + V+   P  +S 
Sbjct  152  GEIESCKLVRDKITGQSLGYGFVNYVDPKDAEKAINTLNGLRLQTKTIKVSYARPSSASI  211

Query  196  R----FTMDLPRDVTPEDIKKVLQPLN--------VDILYNKKLPVGEPNGDKRV  238
            R    +   LP+ +T ++++++             VD +      VG    DKR+
Sbjct  212  RDANLYVSGLPKTMTQKELEQLFSQYGRIITSRILVDQVTGMSRGVGFIRFDKRI  266


>ref|XP_004949594.1| PREDICTED: ELAV-like protein 2 isoform X3 [Gallus gallus]
Length=450

 Score = 40.4 bits (93),  Expect = 6.8, Method: Compositional matrix adjust.
 Identities = 43/175 (25%), Positives = 76/175 (43%), Gaps = 22/175 (13%)

Query  82   SRHPTSYTPWNKSNDKHIASAPLNTNNVSEEKARELCNILIEHLPPQMTKRELKALFNEL  141
            S  PT     + SN  +  S+P+++ N  + K     N+++ +LP  MT+ ELK+LF  +
Sbjct  97   SNGPTCNNTAHGSNAINNCSSPIDSGNTEDSKT----NLIVNYLPQSMTQEELKSLFGSI  152

Query  142  -DIRVNSIV--KIQN---GRAFVALNGEHSLQRILALDGNLTLMQKKVLVTQIGPLKSSW  195
             +I    +V  KI     G  FV        ++ +     L L  K + V+   P  +S 
Sbjct  153  GEIESCKLVRDKITGQSLGYGFVNYVDPKDAEKAINTLNGLRLQTKTIKVSYARPSSASI  212

Query  196  R----FTMDLPRDVTPEDIKKVLQPLN--------VDILYNKKLPVGEPNGDKRV  238
            R    +   LP+ +T ++++++             VD +      VG    DKR+
Sbjct  213  RDANLYVSGLPKTMTQKELEQLFSQYGRIITSRILVDQVTGMSRGVGFIRFDKRI  267


>ref|XP_009965945.1| PREDICTED: ELAV-like protein 2 isoform X1 [Tyto alba]
Length=388

 Score = 40.0 bits (92),  Expect = 7.5, Method: Compositional matrix adjust.
 Identities = 39/144 (27%), Positives = 73/144 (51%), Gaps = 16/144 (11%)

Query  82   SRHPTSYTPWNKSNDKHIASAPLNTNNVSEEKARELCNILIEHLPPQMTKRELKALFNEL  141
            S  PT     + S   +  S+P+N+ N  + K     N+++ +LP  MT+ ELK+LF  +
Sbjct  35   SNGPTCNNTAHSSTTVNNCSSPVNSGNTEDSKT----NLIVNYLPQNMTQEELKSLFGSI  90

Query  142  -DIRVNSIV--KIQN---GRAFV-ALNGEHSLQRILALDGNLTLMQKKVLVTQIGPLKSS  194
             +I    +V  KI     G  FV  ++ +++ + I  L+G L L  K + V+   P  +S
Sbjct  91   GEIESCKLVRDKITGQSLGYGFVNYVDPKNAEKAINTLNG-LRLQTKTIKVSYARPSSAS  149

Query  195  WR----FTMDLPRDVTPEDIKKVL  214
             R    +   LP+ +T ++++++ 
Sbjct  150  IRDANLYVSGLPKTMTQKELEQLF  173


>ref|XP_004949596.1| PREDICTED: ELAV-like protein 2 isoform X5 [Gallus gallus]
Length=449

 Score = 40.0 bits (92),  Expect = 7.5, Method: Compositional matrix adjust.
 Identities = 43/175 (25%), Positives = 76/175 (43%), Gaps = 22/175 (13%)

Query  82   SRHPTSYTPWNKSNDKHIASAPLNTNNVSEEKARELCNILIEHLPPQMTKRELKALFNEL  141
            S  PT     + SN  +  S+P+++ N  + K     N+++ +LP  MT+ ELK+LF  +
Sbjct  97   SNGPTCNNTAHGSNAINNCSSPIDSGNTEDSKT----NLIVNYLPQSMTQEELKSLFGSI  152

Query  142  -DIRVNSIV--KIQN---GRAFVALNGEHSLQRILALDGNLTLMQKKVLVTQIGPLKSSW  195
             +I    +V  KI     G  FV        ++ +     L L  K + V+   P  +S 
Sbjct  153  GEIESCKLVRDKITGQSLGYGFVNYVDPKDAEKAINTLNGLRLQTKTIKVSYARPSSASI  212

Query  196  R----FTMDLPRDVTPEDIKKVLQPLN--------VDILYNKKLPVGEPNGDKRV  238
            R    +   LP+ +T ++++++             VD +      VG    DKR+
Sbjct  213  RDANLYVSGLPKTMTQKELEQLFSQYGRIITSRILVDQVTGMSRGVGFIRFDKRI  267


>ref|XP_009965949.1| PREDICTED: ELAV-like protein 2 isoform X4 [Tyto alba]
Length=387

 Score = 40.0 bits (92),  Expect = 7.8, Method: Compositional matrix adjust.
 Identities = 39/144 (27%), Positives = 73/144 (51%), Gaps = 16/144 (11%)

Query  82   SRHPTSYTPWNKSNDKHIASAPLNTNNVSEEKARELCNILIEHLPPQMTKRELKALFNEL  141
            S  PT     + S   +  S+P+N+ N  + K     N+++ +LP  MT+ ELK+LF  +
Sbjct  35   SNGPTCNNTAHSSTTVNNCSSPVNSGNTEDSKT----NLIVNYLPQNMTQEELKSLFGSI  90

Query  142  -DIRVNSIV--KIQN---GRAFV-ALNGEHSLQRILALDGNLTLMQKKVLVTQIGPLKSS  194
             +I    +V  KI     G  FV  ++ +++ + I  L+G L L  K + V+   P  +S
Sbjct  91   GEIESCKLVRDKITGQSLGYGFVNYVDPKNAEKAINTLNG-LRLQTKTIKVSYARPSSAS  149

Query  195  WR----FTMDLPRDVTPEDIKKVL  214
             R    +   LP+ +T ++++++ 
Sbjct  150  IRDANLYVSGLPKTMTQKELEQLF  173


>ref|XP_004949605.1| PREDICTED: ELAV-like protein 2 isoform X14 [Gallus gallus]
Length=367

 Score = 40.0 bits (92),  Expect = 7.8, Method: Compositional matrix adjust.
 Identities = 43/175 (25%), Positives = 76/175 (43%), Gaps = 22/175 (13%)

Query  82   SRHPTSYTPWNKSNDKHIASAPLNTNNVSEEKARELCNILIEHLPPQMTKRELKALFNEL  141
            S  PT     + SN  +  S+P+++ N  + K     N+++ +LP  MT+ ELK+LF  +
Sbjct  14   SNGPTCNNTAHGSNAINNCSSPIDSGNTEDSKT----NLIVNYLPQSMTQEELKSLFGSI  69

Query  142  -DIRVNSIV--KIQN---GRAFVALNGEHSLQRILALDGNLTLMQKKVLVTQIGPLKSSW  195
             +I    +V  KI     G  FV        ++ +     L L  K + V+   P  +S 
Sbjct  70   GEIESCKLVRDKITGQSLGYGFVNYVDPKDAEKAINTLNGLRLQTKTIKVSYARPSSASI  129

Query  196  R----FTMDLPRDVTPEDIKKVLQPLN--------VDILYNKKLPVGEPNGDKRV  238
            R    +   LP+ +T ++++++             VD +      VG    DKR+
Sbjct  130  RDANLYVSGLPKTMTQKELEQLFSQYGRIITSRILVDQVTGMSRGVGFIRFDKRI  184


>ref|XP_003643117.1| PREDICTED: ELAV-like protein 2 isoformX2 [Gallus gallus]
Length=366

 Score = 40.0 bits (92),  Expect = 7.9, Method: Compositional matrix adjust.
 Identities = 43/175 (25%), Positives = 76/175 (43%), Gaps = 22/175 (13%)

Query  82   SRHPTSYTPWNKSNDKHIASAPLNTNNVSEEKARELCNILIEHLPPQMTKRELKALFNEL  141
            S  PT     + SN  +  S+P+++ N  + K     N+++ +LP  MT+ ELK+LF  +
Sbjct  14   SNGPTCNNTAHGSNAINNCSSPIDSGNTEDSKT----NLIVNYLPQSMTQEELKSLFGSI  69

Query  142  -DIRVNSIV--KIQN---GRAFVALNGEHSLQRILALDGNLTLMQKKVLVTQIGPLKSSW  195
             +I    +V  KI     G  FV        ++ +     L L  K + V+   P  +S 
Sbjct  70   GEIESCKLVRDKITGQSLGYGFVNYVDPKDAEKAINTLNGLRLQTKTIKVSYARPSSASI  129

Query  196  R----FTMDLPRDVTPEDIKKVLQPLN--------VDILYNKKLPVGEPNGDKRV  238
            R    +   LP+ +T ++++++             VD +      VG    DKR+
Sbjct  130  RDANLYVSGLPKTMTQKELEQLFSQYGRIITSRILVDQVTGMSRGVGFIRFDKRI  184


>ref|XP_004949603.1| PREDICTED: ELAV-like protein 2 isoform X12 [Gallus gallus]
Length=375

 Score = 39.7 bits (91),  Expect = 8.5, Method: Compositional matrix adjust.
 Identities = 43/175 (25%), Positives = 76/175 (43%), Gaps = 22/175 (13%)

Query  82   SRHPTSYTPWNKSNDKHIASAPLNTNNVSEEKARELCNILIEHLPPQMTKRELKALFNEL  141
            S  PT     + SN  +  S+P+++ N  + K     N+++ +LP  MT+ ELK+LF  +
Sbjct  22   SNGPTCNNTAHGSNAINNCSSPIDSGNTEDSKT----NLIVNYLPQSMTQEELKSLFGSI  77

Query  142  -DIRVNSIV--KIQN---GRAFVALNGEHSLQRILALDGNLTLMQKKVLVTQIGPLKSSW  195
             +I    +V  KI     G  FV        ++ +     L L  K + V+   P  +S 
Sbjct  78   GEIESCKLVRDKITGQSLGYGFVNYVDPKDAEKAINTLNGLRLQTKTIKVSYARPSSASI  137

Query  196  R----FTMDLPRDVTPEDIKKVLQPLN--------VDILYNKKLPVGEPNGDKRV  238
            R    +   LP+ +T ++++++             VD +      VG    DKR+
Sbjct  138  RDANLYVSGLPKTMTQKELEQLFSQYGRIITSRILVDQVTGMSRGVGFIRFDKRI  192


>ref|XP_004949601.1| PREDICTED: ELAV-like protein 2 isoform X10 [Gallus gallus]
 ref|XP_004949602.1| PREDICTED: ELAV-like protein 2 isoform X11 [Gallus gallus]
Length=388

 Score = 39.7 bits (91),  Expect = 8.6, Method: Compositional matrix adjust.
 Identities = 43/175 (25%), Positives = 76/175 (43%), Gaps = 22/175 (13%)

Query  82   SRHPTSYTPWNKSNDKHIASAPLNTNNVSEEKARELCNILIEHLPPQMTKRELKALFNEL  141
            S  PT     + SN  +  S+P+++ N  + K     N+++ +LP  MT+ ELK+LF  +
Sbjct  35   SNGPTCNNTAHGSNAINNCSSPIDSGNTEDSKT----NLIVNYLPQSMTQEELKSLFGSI  90

Query  142  -DIRVNSIV--KIQN---GRAFVALNGEHSLQRILALDGNLTLMQKKVLVTQIGPLKSSW  195
             +I    +V  KI     G  FV        ++ +     L L  K + V+   P  +S 
Sbjct  91   GEIESCKLVRDKITGQSLGYGFVNYVDPKDAEKAINTLNGLRLQTKTIKVSYARPSSASI  150

Query  196  R----FTMDLPRDVTPEDIKKVLQPLN--------VDILYNKKLPVGEPNGDKRV  238
            R    +   LP+ +T ++++++             VD +      VG    DKR+
Sbjct  151  RDANLYVSGLPKTMTQKELEQLFSQYGRIITSRILVDQVTGMSRGVGFIRFDKRI  205


>ref|XP_004949604.1| PREDICTED: ELAV-like protein 2 isoform X13 [Gallus gallus]
Length=374

 Score = 39.7 bits (91),  Expect = 9.2, Method: Compositional matrix adjust.
 Identities = 43/175 (25%), Positives = 76/175 (43%), Gaps = 22/175 (13%)

Query  82   SRHPTSYTPWNKSNDKHIASAPLNTNNVSEEKARELCNILIEHLPPQMTKRELKALFNEL  141
            S  PT     + SN  +  S+P+++ N  + K     N+++ +LP  MT+ ELK+LF  +
Sbjct  21   SNGPTCNNTAHGSNAINNCSSPIDSGNTEDSKT----NLIVNYLPQSMTQEELKSLFGSI  76

Query  142  -DIRVNSIV--KIQN---GRAFVALNGEHSLQRILALDGNLTLMQKKVLVTQIGPLKSSW  195
             +I    +V  KI     G  FV        ++ +     L L  K + V+   P  +S 
Sbjct  77   GEIESCKLVRDKITGQSLGYGFVNYVDPKDAEKAINTLNGLRLQTKTIKVSYARPSSASI  136

Query  196  R----FTMDLPRDVTPEDIKKVLQPLN--------VDILYNKKLPVGEPNGDKRV  238
            R    +   LP+ +T ++++++             VD +      VG    DKR+
Sbjct  137  RDANLYVSGLPKTMTQKELEQLFSQYGRIITSRILVDQVTGMSRGVGFIRFDKRI  191


Lambda      K        H        a         alpha
   0.313    0.132    0.381    0.792     4.96 

Gapped
Lambda      K        H        a         alpha    sigma
   0.267   0.0410    0.140     1.90     42.6     43.6 

Effective search space used: 7359541678981


  Database: nr
    Posted date:  Sep 23, 2015 12:05 AM
  Number of letters in database: 26,053,659,533
  Number of sequences in database:  71,551,133


Matrix: BLOSUM62
Gap Penalties: Existence: 11, Extension: 1
Neighboring words threshold: 11
Window for multiple hits: 40
```
